# Characteristics of *Burkholderia pyrrocinia* and the biocontrol mechanism by which it affects lily bulb rot caused by *F. oxysporum*

**DOI:** 10.3389/fmicb.2026.1788193

**Published:** 2026-04-23

**Authors:** Nana Chang, Gao Zhou, Yan Ren, Chu Wang, Ye Cao, Lingling Zheng, Ye Wang, Ying Zeng

**Affiliations:** 1 Jiangxi Province Key Laboratory of Sustainable Utilization of Traditional Chinese Medicine Resources, Institute of Traditional Chinese Medicine Health Industry, China Academy of Chinese Medical Sciences, Nanchang, China; 2 Jiangxi Health Industry Institute of Traditional Chinese Medicine, Nanchang, China

**Keywords:** antagonistic activity, biocontrol, bulb rot, *Burkholderia pyrrocinia*, *Fusarium oxysporum*, metabolomics, transcriptomics

## Abstract

**Background:**

Lily bulb rot, which is mainly caused by *Fusarium oxysporum* (*F. oxysporum*), is a destructive soil-borne disease that significantly reduces lily yield and quality. Biocontrol strategies are preferable for counteracting the threats associated with pathogens.

**Methods:**

Confrontation assays were used to screen antagonistic rhizosphere bacteria against *F. oxysporum* and evaluate their antifungal activity. The most effective isolate, *Burkholderia pyrrocinia*, was further characterized using a multi-omics approach to investigate its modulation of lily defense responses.

**Results:**

In this study, the strain YFLYS026, identified as *B. pyrrocinia*, was isolated from the rhizosphere soil surrounding healthy lily bulbs. It not only exhibited distinct antagonistic activity against six phytopathogens, with inhibition rates on PDA plates ranging from 46.62 to 59.02%, but also showed plant growth-promoting traits, such as nitrogen fixation, phosphorus solubilization, and siderophore production. In pot experiments, strain YFLYS026 effectively controlled bulb rot in lilies, showing a biocontrol efficacy of 61.11%. A total of 1,592 differentially expressed genes (DEGs) were shared in both treatment groups compared with the control; most of these genes were upregulated, and their composition indicated rapid activation of the plant immune network. The DEGs shared between the two treatment groups were significantly enriched in flavonoid, phenylpropanoid, starch/sucrose and alkaloid metabolic pathways associated with energy production and defense compound biosynthesis. Meanwhile, we found that strain YFLYS026 inoculation upregulated dirigent protein (*DIR*) expression, driving pinoresinol accumulation and cell wall lignification to form a physical barrier against pathogen invasion. Integrated multi-omics analysis suggested that phenylpropanoid and flavonoid metabolic pathways were downregulated, potentially indicating carbon metabolic flux redistribution to prioritize lignin synthesis.

**Discussion:**

These results support the potential of *B. pyrrocinia* YFLYS026 to alleviate lily bulb rot disease and lay a foundation for its application as an effective biocontrol agent.

## 1 Introduction

Lily (*Lilium brownii* var. *viridulum*), commonly known as Longya lily, is an economically significant bulbous flower crop within the Liliaceae family (Tu et al., 2023). This species exhibits exceptional ornamental, nutritional, and medicinal value, with bulbs containing diverse secondary metabolites, including phenols, saponins, sterols, and alkaloids, that demonstrate sedative, anti-inflammatory, and antitussive activities (Tang et al., 2021). Lily has been cultivated for over 500 years; today, it is cultivated mainly in Wanzai County, Jiangxi Province, one of the three major lily production areas in China, where Baishui Township is considered the “Hometown of Lily” (Yang et al., 2019). However, as the cultivation areas expand and consecutive cropping years increase, the prevalence of lily diseases has also increased. In particular, bulb rot, a disease primarily caused by *Fusarium* spp., has an incidence of approximately 30%, which in severe cases can exceed 60%; this disease significantly affects lily cultivation and production (Jiao et al., 2021; Tu et al., 2023). The disease begins with brown, slightly concave spots on bulb scales during the early developmental stages; these spots gradually expand and undergo necrosis, ultimately resulting in complete bulb decay, scale detachment from the basal plate, leaf wilting, and plant mortality (Jiao et al., 2021). Disease progression is further influenced by environmental factors, including temperature fluctuations, soil moisture content, soil compaction, and drainage conditions, that create favorable conditions for pathogen establishment and proliferation. Traditionally, the control of lily bulb rot has relied mainly on the use of various chemical pesticides (Jiang et al., 2021). Although such chemical control is effective, its extensive use not only leads to soil and water pollution-threatening food safety and thus possibly harming human health directly, but also may result in drug resistance in the responsible pathogens (Luo et al., 2023; Naqvi et al., 2025). To reduce the dependence on chemical products in agricultural practice, there has been growing emphasis on the adoption of biological control measures for disease management.

Biological control, defined as the purposeful use of beneficial microorganisms to suppress plant pathogens through a variety of mechanisms, including direct antagonism, competition for resources, and induction of host plant defenses, has been recognized as an effective strategy for improving agricultural sustainability (Fontana et al., 2021; Das et al., 2024). Similarly, the use of biological control agents (BCAs) in disease management, such as *Bacillus* spp. (Tu et al., 2023), *Burkholderia* spp. (Gong et al., 2019; Heo et al., 2022), and *Pseudomonas* spp. (Huang et al., 2025), has achieved notable success against pathogens that cause various plant diseases. *Burkholderia* spp., Gram-negative bacteria that are widely distributed across natural environments, including soil, water, and plant rhizospheres, are plant endophytes that have recently garnered increased scientific interest (Wang et al., 2023). This genus is markedly diverse and includes nitrogen-fixing members that provide essential nitrogen to plants, promote plant growth, and increase the production of defense compounds in plants that improve resistance to phytopathogens (Liu et al., 2022). Multiple *Burkholderia* spp. have demonstrated potential as BCAs and biofertilizers in agricultural applications, such as *B. cepacia* (Zhao et al., 2014), *B. contaminans* (Zaman et al., 2021), and *B. pyrrocinia* (Zhu et al., 2024). Specifically, these species establish beneficial relationships with plants through two mechanisms: promoting plant growth and inhibiting phytopathogens via the production of antimicrobial compounds. Key bioactive metabolites of these species include pyochelin, pyrrolnitrin, burkholdine, and cepacin (Bach et al., 2022; Ho et al., 2021; Zaman et al., 2021). Among these species, *B. pyrrocinia* has shown significant promise in reducing the incidence of disease while preserving fruit quality. The *B. pyrrocinia* strain JK-SH007 effectively inhibits poplar canker through the production of volatile organic compounds (VOCs) (Liu et al., 2020). Similarly, the *B. pyrrocinia* strain BX1 demonstrated a remarkable ability to combat banana anthracnose while preserving fruit quality by inhibiting both the growth and appressorium formation of *Colletotrichum fructicola* (Zhu et al., 2024). Recent genomic analyses of *B. pyrrocinia* strains have revealed extensive clusters of secondary metabolite biosynthetic genes, including those responsible for pyrrolnitrin production, siderophore biosynthesis, and nonribosomal peptide synthesis (Esmaeel et al., 2016; Zhang et al., 2025). While previous studies have demonstrated the general antifungal activity of *Burkholderia* spp., particularly *B. pyrrocinia*, against various phytopathogens, the underlying molecular mechanisms governing these antagonistic relationships remain inadequately characterized. Understanding the specific mechanisms by which *B. pyrrocinia* controls lily bulb rot is crucial for the development of effective biocontrol strategies.

In this study, an antagonistic strain, *B. pyrrocinia* YFLYS026, was isolated and identified from the rhizosphere soil of healthy lily bulbs. It exhibited good antifungal activity against various pathogens, including *Fusarium* spp., *C. fructicola*, and *Alternaria alternata*, both *in vitro* and in pot experiments. In addition, multiomics analysis was used to preliminarily elucidate the biocontrol mechanisms of this strain to lily bulb rot. These findings not only advance our understanding of *Burkholderia*-mediated biocontrol but also facilitate the optimization of biocontrol applications and contribute to the advancement of environmentally friendly agriculture.

## 2 Materials and methods

### 2.1 Isolation, identification and pathogenicity testing of pathogenic fungi causing lily root rot

Diseased lily bulbs were collected from the plantation area in Wanzai County, Jiangxi Province, in August 2024. The bulbs were surface disinfected using 70% ethanol for 1 min, then treated with a 0.3% sodium hypochlorite solution for 3 min. Afterward, the bulbs were rinsed five times with sterilized water to thoroughly eliminate any remaining sterilizing agent. The diseased tissues adjacent to asymptomatic regions were cut into small pieces and placed on potato dextrose agar (PDA) supplemented with chloramphenicol (20 μg mL^−1^) (Huankai Microbial, Guangdong, China). The plates were incubated at 28 °C until hyphae emerged from the necrotic tissue. Single hyphal tips were then picked from the necrotic tissue with a sterilized inoculation needle and transferred onto PDA for further incubation at 28 °C to obtain pure culture isolates. After 5–7 days of cultivation, the colonies were preliminarily identified to the species level on the basis of colony characteristics and the morphology of mycelia and conidia observed microscopically. To confirm the identification, the fungal internal transcribed spacer (ITS) gene was amplified with primers ITS1/ITS4, the translation elongation factor 1-*α* (*tef-1α*) gene was amplified with primers EF-1/EF-2, and the RNA polymerase second largest subunit (*RPB2*) gene was amplified with primers RPB2-5f2/RPB2-7cr (O’Donnell et al., 2015). ITS, *TEF* and *RPB2* sequences were aligned with the sequences in the NCBI database with nucleotide BLAST (https://blast.ncbi.nlm.nih.gov/) to obtain reference strain sequences with high similarity. To further assess phylogenetic relationships, a joint phylogenetic tree was constructed using the maximum likelihood method with 1,000 bootstrap replicates in MEGA-X on the basis of the ITS, *TEF* and *RPB2* genes (Supplementary Table S1) and *Fusarium phyllophilum* (CBS 216.76) served as the outgroup.

To verify the pathogenicity of the identified fungal species, 1-year-old lily bulbs were inoculated with mycelia from injurious rhizomes. A fine needle was used to puncture the middle of each fresh scale. Separately, a 6 mm diameter mycelial disc was excised from a 7-day-old culture of the root rot pathogenic fungus and placed onto the wounded scale, ensuring that the hyphal side was in contact with the wound. The inoculated bulbs were then placed in a sterile Petri dish with two pieces of moistened filter paper. The plates were subsequently incubated in a light incubator at 25 °C for one week, alternating between 12 h of light and 12 h of darkness. Blank PDA discs were included in the study as negative controls. Three scales were used for each treatment, and symptoms were observed daily.

### 2.2 Isolation and screening of antagonistic strains from soil

To obtain antagonistic strains of lily bulb fungal pathogens, cultivable bacteria were isolated from healthy lily rhizosphere soil. Each soil sample (1 g) was suspended in 9 mL of sterile distilled water and shaken for 10 min. The solution was then serially diluted 10-fold to 10^−7^. A 100 μL aliquot of the diluted soil was plated on tryptic soy agar (TSA; Becton, Dickinson and Co., Sparks, MA, United States) plates, which were subsequently incubated at 30 °C for 1–3 days. A single colony was then isolated and incubated in LB liquid medium at 30 °C with constant shaking at 180 rpm for 1–2 days and then stored at −80 °C in 30% glycerol until use. Inoculate the antagonistic bacteria onto blood agar plates (Huankai Microbial, Guangdong, China), and place it in a culture box for 24 h (30 °C) to evaluate its safety. The antifungal activity of these strains was later assessed on PDA plates using plate confrontation experiments and *F. oxysporum*-induced bulb rot as indicators. At least three biological replicates were performed.

### 2.3 Identification of the strain YFLYS026

#### 2.3.1 Morphological observations

A total of 15 antagonistic strains were isolated, among which YFLYS026 exhibited the strongest inhibitory activity against pathogenic fungi. Therefore, we selected this strain for further research. YFLYS026 was cultured on TSA agar medium, and the colony morphology was observed under a microscope and by Gram staining.

#### 2.3.2 Molecular identification

To obtain the molecular identity of YFLYS026, the strain was inoculated into LB liquid medium and incubated overnight at 28 °C with shaking at 180 rpm. The bacterial cells were collected by centrifugation at 7200 rpm. Genomic DNA was extracted using the CTAB method, and the 16S rDNA and DNA gyrase subunit B (*gyrB*) were amplified using the primers 27F/1492R and UP-1/UP-2r, respectively (Aulia Rahma et al., 2020). The amplified products were sent to Majorbio Co., Ltd. (Shanghai, China) for sequencing, and the results were analyzed using the BLAST tool from NCBI. The sequencing results were uploaded to GenBank. A data set of 14 *Burkholderiaceae* strains, including the YFLYS026 strain with the 16S rDNA and *gyrB* sequences, was obtained from the GenBank database and aligned with MAFFT (v7.505) (Katoh and Standley, 2013) using the ‘--auto’ strategy and normal alignment mode. The alignments of selected strains were concatenated into a supermatrix using a custom script. Maximum likelihood reconstruction was performed in IQ-TREE (v3.0.1) with default parameters and 1,000 bootstrap replicates. *Paraburkholderia phymatum* (STM815) was used as the outgroup. The phylogenetic tree and support values were visualized using FigTree (v1.4.2).

### 2.4 Assessment of the antagonistic spectrum of strain YFLYS026 *in vitro*

To assess the antagonistic spectrum of strain YFLYS026, its inhibitory effect against 6 plant pathogenic fungal strains was further evaluated through plate confrontation experiments. The indicator strains (*F. oxysporum*, *Fusarium commune*, *Fusarium annulatum*, *Fusarium solani*, *Alternaria alternata* and *Colletotrichum fructicola*) were isolated from diseased lily bulbs, *Artemisia stolonifera* (Maxim.) Komar. leaves, and *Turpinia arguta Seem*. leaves by our laboratory. Strain YFLYS026 was cultured in LB liquid medium at 30 °C with shaking at 180 rpm for 24 h. The cells were harvested, washed twice with PBS, and then resuspended in PBS to an optical density (OD_600nm_) of 1.0. For each pathogenic fungus, a 6 mm mycelial plug from a 5-day-old specimen was positioned in the center of a PDA plate, while a 5 μL aliquot of the YFLYS026 cell suspension (OD_600nm_ = 1.0) was applied in a straight line on both sides of the mycelial plug at a distance of 20 mm. The plates were inverted and incubated at 28 °C for 5 days. Plates with only a fungal plug served as controls, and each test was conducted in three replicates. The colony diameters of the cultured pathogens were recorded, and the inhibition rate was calculated using the following formula.


$$\begin{array}{l} \text{Inhibition rate}\;(\%)=\\ \frac{\text{control colony diameter}-\text{bacteria treatment colony diameter}}{\text{control colony diameter}}\times 100 \end{array}$$

### 2.5 Effect of YFLYS026 VOCs on hyphal growth of *F. oxysporum*

The double-plate assay was employed to evaluate the antifungal activity of YFLYS026 against *F. oxysporum* (Yin et al., 2023). YFLYS026 was cultured in LB liquid medium at 30 °C with shaking at 180 rpm for 24 h. The bacterial suspension was then adjusted to OD₆₀₀ = 1.0, and 100 μL was evenly spread onto LB agar medium using a sterile spreader. A 6 mm plug from a 5-day-old culture of the pathogenic fungus was placed at the center of a fresh PDA plate. The LB plate (containing YFLYS026) and the PDA plate (containing the fungal plug) were sealed together with Parafilm with the PDA plate inverted on top, and incubated at 28 °C for 7 days. As a negative control, LB agar spread with 100 μL of sterile liquid LB (without bacteria) was paired with a PDA plate containing the fungal plug.

### 2.6 Pot experiment for assessing the biocontrol potential of strain YFLYS026

The mixture of nutrient soil, vermiculite, and perlite in a volume ratio of 3:1:1 was sterilized at 121 °C for 30 min. Tissue-cultured lilies that had been grown for two weeks at 25 ± 1 °C were transplanted into plastic pots and cultivated outdoors for another two weeks before they were assessed for lily root rot. All the plants were dug up, and their bulbs were wounded with a sterile needle before being retransplanted into the pots. Healthy plants were next treated by adding the following to the soil surrounding each bulb: (control, CK): 20 mL of distilled water; (treatment 1, T1): 10 mL of ddH_2_O and 10 mL of *F. oxysporum* spore suspension (1 × 10^6^ spores/mL); and (treatment 2, T2): 10 mL of YFLYS026 suspension (OD_600nm_ = 1.0) and 10 mL of *F. oxysporum* spore suspension (1 × 10^6^ spores/mL). All the plants were grown in an artificial climate incubator at 28 °C and 80% relative humidity with a 12 h photoperiod for 7 days. Disease severity was scored on a 0–3 scale based on observations of leaf chlorosis and bulb rot symptoms: 0: normal growth; 1: less than 25% of the plant leaf chlorosis; 2: 25–75% of the plant leaf chlorosis with slight browning of the bulb; 3: greater than 75% of the whole plant had wilted or died. Each group comprised six healthy lily plants and three biological replicates. Disease index and biocontrol efficiency were calculated by the following formulas.


$$\text{Incidence rate}\;(\%)=\frac{\text{number of infected plants}}{\text{total number of plants}}\times 100$$

$$\text{Disease index}\;(\%)=\sum \frac{\text{grade number of infected plants}}{\text{highest grade}\times \text{total number of}\;\;\text{plants}}\times 100$$

$$\begin{array}{l} \text{Biocontrol efficiency}\;(\%)=\\ \frac{\text{disease index of control}-\text{disease index of treatment}\;}{\text{disease index of control}}\times 100 \end{array}$$

### 2.7 Transcriptome sequencing of lilium bulbs treated with strain YFLYS026

To analyze the resistance mechanisms induced by strain YFLYS026 in lilies, bulb tissues were collected from lily plants seven days after different treatments, with three biological replicates per treatment. Each replicate consisted of mixed bulb samples from six lily plants, which were quick-frozen with liquid nitrogen and stored at −80 °C. Then, all frozen samples were sent on dry ice to MetWare Biotechnology Co., Ltd. (Wuhan, China) for transcriptome sequencing. To estimate changes in gene expression between the control and treated samples, FASTP was used to filter the raw RNA-seq data to remove low-quality and adapter-contaminated reads. Clean reads were assembled *de novo* using Trinity (https://github.com/trinityrnaseq/trinityrnaseq) without a reference genome, and the assembled transcripts were clustered and de-redundant using Corset (https://github.com/Oshlack/Corset/wiki/Example). Assembly quality was assessed based on total sequences and bases, GC content, unigene length distribution, average coverage, and N50/N90 statistics. Transcriptome assembly completeness was assessed using software BUSCO based on alignments to the Benchmarking Universal Single-Copy Orthologs database. The RNA-seq reads of each sample were then quantified, and their abundance (transcripts per million) was calculated. After redundant sequences were removed, the transcripts were aligned with sequences in the Kyoto Encyclopaedia of Genes and Genomes (KEGG) and evolutionary genealogy of genes: Nonsupervised Orthologous Groups (eggNOG) databases using DIAMOND software. The expression level of the transcripts was calculated using RSEM software, after which the FPKM of each transcript was calculated according to the transcript length. DESeq2 software was used to compute the differential expression between the two groups and identify differentially expressed genes (DEGs) with a false discovery rate (FDR) < 0.05 and |log_2_Fold Change| ≥ 1. The DEGs were used for subsequent KEGG pathway enrichment analysis. Weighted gene co-expression network analysis (WGCNA) was applied to identify hub genes and core metabolites significantly associated with disease resistance with R software (Langfelder and Horvath, 2008). The sequencing information was deposited in the NCBI database under accession number PRJNA1143388. Quantitative reverse-transcription polymerase chain reaction (RT-qPCR) was used to detect the expression patterns of the DEGs obtained from the RNA-seq analysis. The relative gene expression values were calculated using the 2^−ΔΔCt^ method with *β*-actin as the internal reference gene (Schmittgen and Livak, 2008); all primers used are listed in Supplementary Table S2.

### 2.8 Metabolomics analysis of lilium bulbs treated with strain YFLYS026

The transcriptome samples were further subjected to metabolite extraction. Using vacuum freeze-drying technology, the tissue samples were placed in a lyophilizer (Scientz-100F), after which they were ground (30 Hz, 1.5 min) into powder form with a grinder (MM 400, Retsch). Next, 30 mg of sample powder was weighed using an electronic balance (MS105DU), and 1,500 μL of 70% methanolic aqueous internal standard extract precooled to −20 °C was added (less than 30 mg at a rate of 1,500 μL extractant per 30 mg sample). The mixture was vortexed once every 30 min for 30 s for a total of 6 times. After centrifugation at 12000 rpm for 3 min, the supernatant was collected, and the sample was filtered through a microporous membrane (0.22 μm pore size) and stored in an injection vial for ultraperformance liquid chromatography–tandem mass spectrometry (UPLC-MS/MS) analysis. The UPLC analytical conditions were as follows: column, Agilent SB-C18 (1.8 μm, 2.1 mm × 100 mm); and mobile phase, solvent A (pure water with 0.1% formic acid) and solvent B (acetonitrile with 0.1% formic acid). Gradient elution was performed as follows: starting conditions, 95% A and 5% B; linear gradient to 5% A and 95% B over 9 min, which was then maintained for 1 min; and a return to 95% A and 5.0% B over 1.1 min, which was then maintained for 2.9 min. The flow rate was set to 0.35 mL per minute, the column oven was maintained at 40 °C, and the injection volume was 2 μL. The effluent was alternatively connected to an ESI-triple quadrupole-linear ion trap (QTRAP) mass spectrometer. The ESI source operation parameters were as follows: source temperature, 500 °C; ion spray voltage, 5.5 kV (positive mode) and 4.5 kV (negative mode); ion source gas I, gas II, and curtain gas, 50, 60, and 25 psi, respectively; collision-activated dissociation, high; and collision gas (nitrogen), medium. Analyst software (Version 1.6.3, AB Sciex, Framingham, MA, United States) was used for data analysis. Metabolites were qualitatively analyzed on the basis of secondary spectral information from the custom MetWare database (MWDB) and quantitatively analyzed using triple quadrupole mass spectrometry multiple reaction monitoring (MRM) mode. Multivariate analysis of the identified metabolites was performed by principal component analysis (PCA). Metabolites with |log_2_Fold Change| ≥ 1 and a VIP (variable importance in projection) > 1 were considered differentially accumulated metabolites (DAMs). The DAMs were subsequently annotated using the KEGG Compound database (http://www.kegg.jp/kegg/compound/), and the annotated metabolites were mapped to the KEGG Pathway database to identify enriched metabolic pathways.

### 2.9 Combined transcriptome and metabolome analysis

On the basis of the RNA-seq data, we used weighted gene co-expression network analysis (WGCNA) to identify gene modules associated with resistance against *F. oxysporum*. After background correction and normalization, a soft threshold power of *β* = 12 was selected to achieve scale-free topology. Genes with similar expression patterns were clustered into modules, and module eigengenes (MEs) were calculated. All DAMs mapped to the top 10 most enriched metabolic pathways (ranked by rich factor and q-value) were defined as core metabolites. Pearson correlation analysis was performed between MEs and these core metabolites (|r| > 0.8, *p* < 0.01), and the module showing the strongest correlation was identified as the key module. KEGG pathway enrichment analysis was conducted for genes within this key module. The enrichment results and correlation networks were visualized using Cytoscape (version 3.5.1) and Adobe Illustrator, respectively.

### 2.10 Statistical analyses

GraphPad Prism 9.0 and IBM SPSS v30 were used for graphing and statistical analysis, respectively. Two-group data were analyzed by two-tailed unpaired t-test; multi-group data by one-way ANOVA followed by Duncan’s test. *p* < 0.05 was set as the significance level (*p < 0.05, **p < 0.01, ***p < 0.001).

## 3 Results

### 3.1 Identification and characterization of lily bulb pathogenic fungi

**Figure 1:**
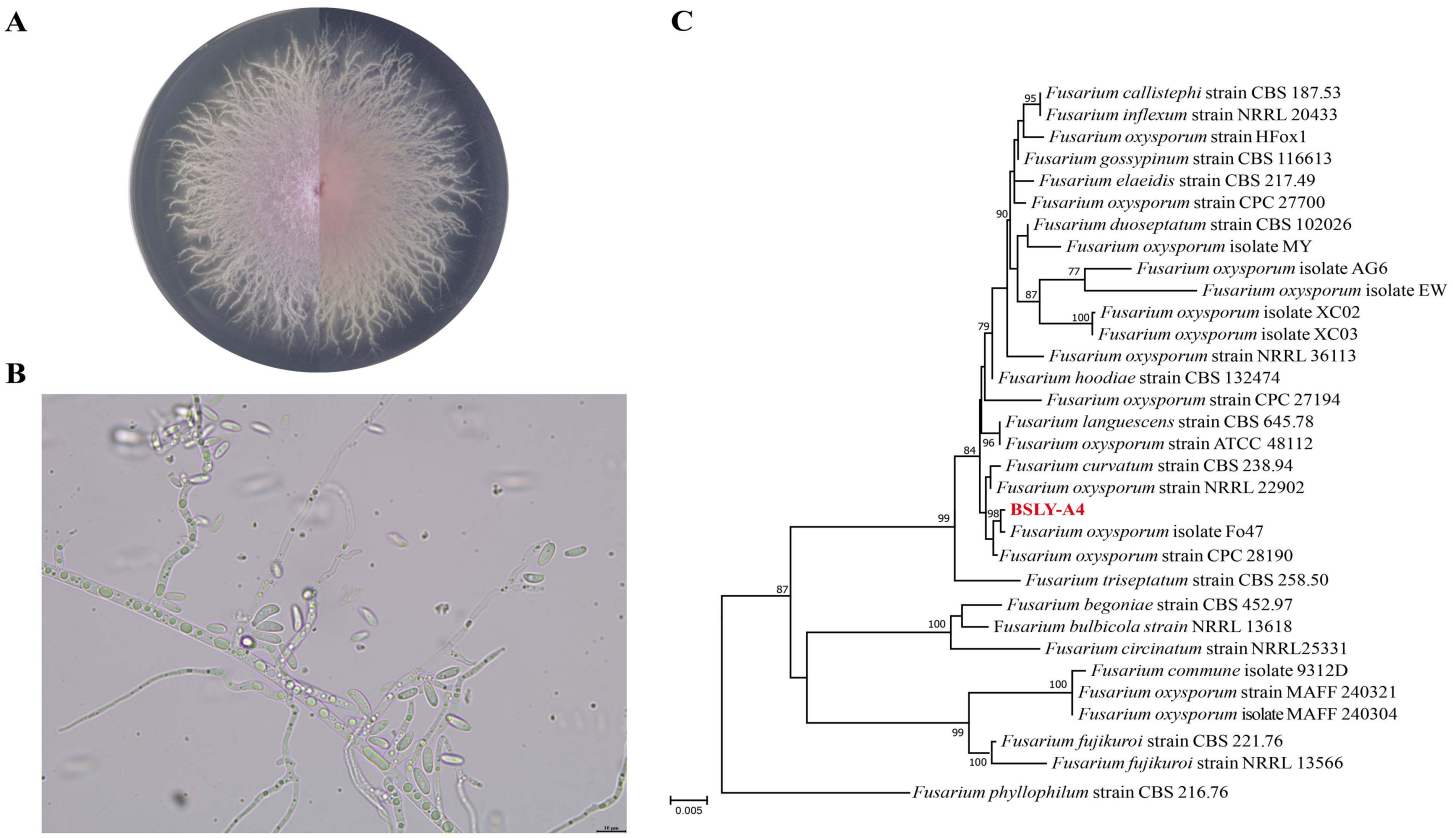
**(A)** Colony features of strain BSLY-A4 on PDA medium. **(B)** The spore morphology of strain BSLY-A4. **(C)** Phylogenetic tree based on DNA-ITS/*tef*-1α /*RPB2* combined sequences of strain BSLY-A4.

According to field investigations, the diseased lily plants exhibited yellowing leaves, bulb rot, and stunted growth, ultimately resulting in wilting and death, which severely affected yield (Supplementary Figures 1A–C). In this study, lily bulbs with leaf wilt and even plant death were collected for pathogen isolation from two lily cultivation areas, the towns of Baishui and Sanxing in Wanzai County. Pathogenic fungi were isolated and purified from diseased lily bulbs using the tissue isolation method. Thirty-two purified fungal isolates were obtained, of which eighteen appeared to be *Fusarium* spp. (isolation rate of 56.3%). The surfaces of the eighteen fungi were white with dense aerial mycelia. Some had an orange or purple center in the middle. The microconidia were oval in shape and appeared either straight or slightly curved. Cells at the apex and base were similarly curved. To confirm the infectivity of these strains, eighteen isolates were selected for pathogenicity tests. The results showed that eight strains (BSLY-A1, BSLY-A4, BSLY-A10, YFLYH-D1-2, YFLYH-C3, YFLYH-C3-2, YFLYH-E3 and YFLYH-E3-1) caused symptoms on the wounded inoculated bulb sites (Supplementary Figures 2, 3A), indicating that these strains might enter through wounds to cause bulb rot. Based on a sequence comparison of the internal transcribed spacer (ITS) region, isolated strains were divided into three different clusters: cluster I (BSLY-A4 and YFLYH-D1-2) belonged to *F. oxysporum*, cluster II (YFLYH-C3, YFLYH-C3-2, YFLYH-E3 and YFLYH-E3-1) was grouped with *F. fujikuroi*/*F. annulatum*, and cluster III (BSLY-A1 and BSLY-A10) belonged to *F. commune* (Supplementary Figure 3B). Previous studies have reported that the pathogenic fungus causing bulb rot is predominantly *F. oxysporum* on the basis of ITS molecular identification (Jiao et al., 2021). Therefore, the sequences of the *tef-1α* and *RPB2* genes from *F. oxysporum* BSLY-A4 were selected for amplification to confirm the identity of the pathogen. These sequences were submitted to GenBank (ITS: PX232664; *TEF*: PX244388; *RPB2*: PX259951). A joint phylogenetic tree was constructed based on the ITS, *TEF* and *RPB2* genes to further assess the phylogenetic relationships. According to the results of the morphological identification and phylogenetic tree analysis, the isolate BSLY-A4 was identified as *F. oxysporum* (Figures 1A–C).

### 3.2 Isolation and screening of antagonistic bacteria

To identify antagonistic bacteria that can prevent bulb rot caused by *F. oxysporum*, cultivable bacteria were isolated from the rhizosphere soil of healthy lily bulbs. Through isolation and purification, a total of 211 isolates were determined, and the corresponding 16S rRNA gene sequences were obtained using PCR amplification followed by sequencing. Comparison of the 16S rRNA gene sequences using the EzBioCloud database revealed that these isolates belong to the phyla *Proteobacteria*, *Actinobacteria*, and *Firmicutes*. Among them, the dominant genera included *Bacillus* (41.71%), *Paenibacillus* (9.48%), *Priestia* (8.06%), *Burkholderia* (7.11%), and *Arthrobacter* (7.11%) (Supplementary Figure 4A). To evaluate the antifungal activity of these strains and their VOCs, *F. oxysporum* BSLY-A4 was used as an indicator strain for validation. We found that a strain designated YFLYS026 and its VOCs effectively inhibited the mycelial growth of *F. oxysporum*, with a mycelium inhibition rate of 53.45 and 23.10% compared with that of the control group, respectively (Supplementary Figure 4B), indicating that the antifungal activity was primarily attributed to antimicrobial metabolites rather than VOCs.

### 3.3 Morphological, physiological, and biochemical characteristics of the YFLYS026 strain

**Figure 2:**
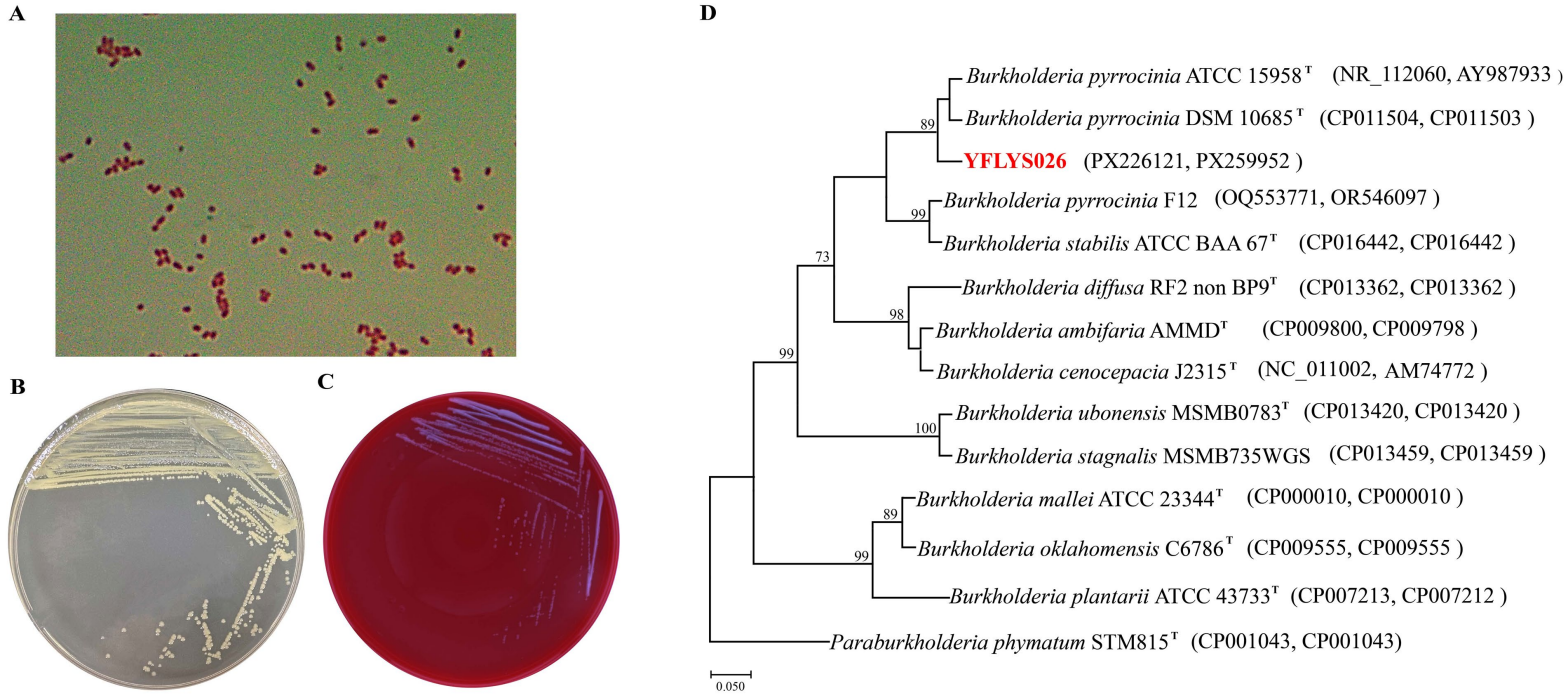
**(A)** Gram staining of strain YFLYS026. **(B)** Colony features of strain YFLYS026 on TSA medium. **(C)** Hemolytic activity assay. **(D)** Joint phylogenetic tree of strain YFLYS026 based on 16S rRNA and *gyrB* gene sequences. *Paraburkholderia phymatum* STM815 strain was used as the outgroup. Bootstrap values greater than 70% are shown at branch points. “T” stands for type strains. The GenBank accession numbers are given in parentheses after the strain name.

Strain YFLYS026 was Gram-negative with short rod-shaped cells, and The YFLYS026 colonies grown on TSA plates appeared light yellow, circular, smooth, and had neat edges (Figures 2A,B). Hemolytic activity assay showed that the YFLYS026 strain produced no hemolytic zone on blood agar plates, preliminarily suggesting no potential biosafety risk for agricultural application (Figure 2C). The predominant cellular fatty acids of strain YFLYS026 were C_16:0_ (24.86%), C_17:0_ cyclo (16.48%), C_16:0_ 3OH (5.56%), and C_19:0_ cyclo ω8c (4.22%) (Supplementary Table S3). Strain YFLYS026 was able to dissolve inorganic phosphorus, fix nitrogen, and produce siderophores (Supplementary Figures 5A–C), indicating that it might promote plant growth. Furthermore, multigene phylogenetic analysis performed based on 16S rRNA and *gyrB* genes with *Paraburkholderia phymatum* STM815 as the outgroup showed the YFLYS026 strain within the *B. pyrrocinia* clade (Figure 2D). On the basis of its culture, morphological, physiological, biochemical, and genetic characteristics, YFLYS026 was identified as *B. pyrrocinia*. The 16S rRNA and *gyrB* gene sequences of strain YFLYS026 were deposited in GenBank (16S rRNA, PX226121; *gyrB*, PX259952). The strain was subsequently deposited in the China General Microbiological Culture Collection Center (CGMCC) as CGMCC No. 35236.

### 3.4 Antifungal spectrum of YFLYS026 cell suspensions

To evaluate the antifungal activity of strain YFLYS026 against plant pathogenic fungi, antagonism tests were conducted using *F. oxysporum, F. commune*, *F. annulatum*, *F. solani*, *A. alternata* and *C. fructicola* as indicator strains. The results revealed varying degrees of antifungal activity, with inhibition rates ranging from 46.62 to 59.02%. YFLYS026 exhibited the highest inhibition rate against *C. fructicola* (59.02%), followed by *A. alternata* (53.53%) and *F. oxysporum* (53.45%), with relatively lower rates against *F. solani* (46.62%), *F. annulatum* (48.94%), and *F. commune* (49.94%) (Supplementary Figures 6A,B). These findings indicated that the YFLYS026 strain derived from the rhizosphere soil of lily has the potential for broad-spectrum inhibition of plant pathogenic fungi and could be used for the biological control of lily bulb rot.

### 3.5 Disease control efficacy of strain YFLYS026 against lily bulb rot in pot experiments

**Figure 3:**
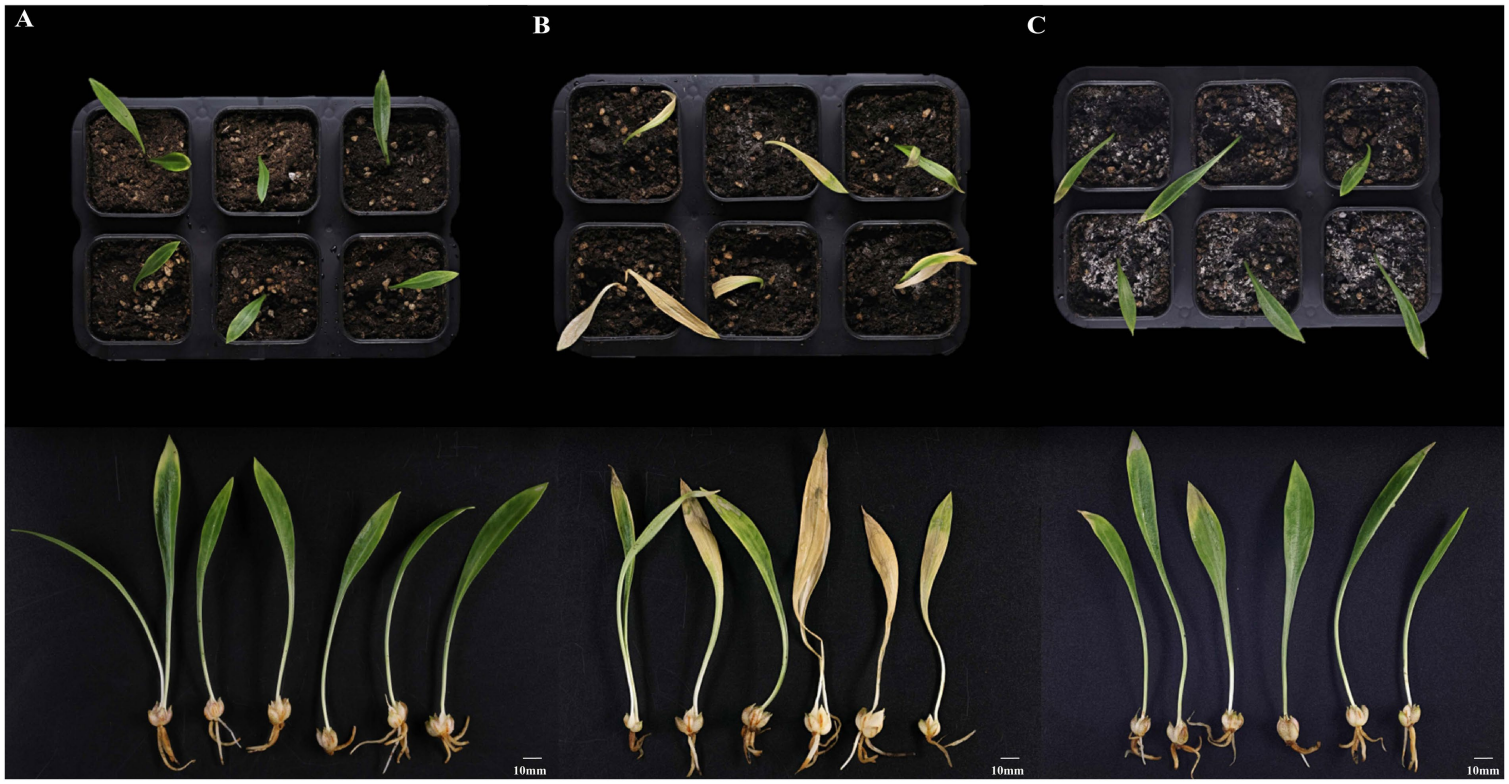
**(A)** Lily plants treated with distilled water: CK. Scale bar = 10 mm. **(B)** Lily plants treated with distilled water and *F. oxysporum* spore suspension: T1. Scale bar = 10 mm. **(C)** Lily plants treated with the mixture of YFLYS026 suspension and *F. oxysporum* spore suspension: T2. Scale bar = 10 mm.

To evaluate the biocontrol potential of strain YFLYS026 against lily bulb rot, pot experiments were conducted under controlled greenhouse conditions. The results revealed significant differences in plant health and disease development among the three groups. In the CK group, all lily plants maintained healthy growth throughout the experimental period. Specifically, the aerial parts of the lily plants remained green and healthy, and the bulbs remained white and firm without any signs of browning or decay. Moreover, the root systems were well developed with robust root hairs, indicating normal nutrient and water absorption (Figure 3A). In comparison, the infected group (T1) exhibited severe disease symptoms. The above-ground tissues showed pronounced yellowing, wilting, and stunted growth, and the bulbs developed conspicuous brown lesions and evidence of rot at sites where the bulb was wounded and the pathogen suspension was inoculated (Figure 3B). Remarkably, the biocontrol treatment group (T2) maintained relatively healthy growth, the bulbs remained largely free from browning and decay, and the structural integrity of the root systems was preserved with healthy root hairs (Figure 3C). Statistical analysis (Table 1) showed that strain YFLYS026 significantly reduced the disease incidence of lily under greenhouse conditions, with a biocontrol efficacy of 61.11%.

**Table 1:** The biocontrol efficacy of strain YFLYS026 in greenhouse.

Treatment group	Incidence rate (%)	Disease index (%)	Biocontrol efficacy (%)
T1 (*F. oxysporum*)	100 ± 0 a	66.67 ± 0.81 a	/
T2 (*F. oxysporum+*YFLYS026)	44.44 ± 2.39 b	25.93 ± 1.56 b	61.11 ± 1.2

Data in the table are mean ± standard deviation (SD). Different lowercase letters in the same column indicated a significant difference between the treatments (*p* < 0.05).

### 3.6 Transcriptome analysis of lily bulbs treated with strain YFLYS026

**Figure 4:**
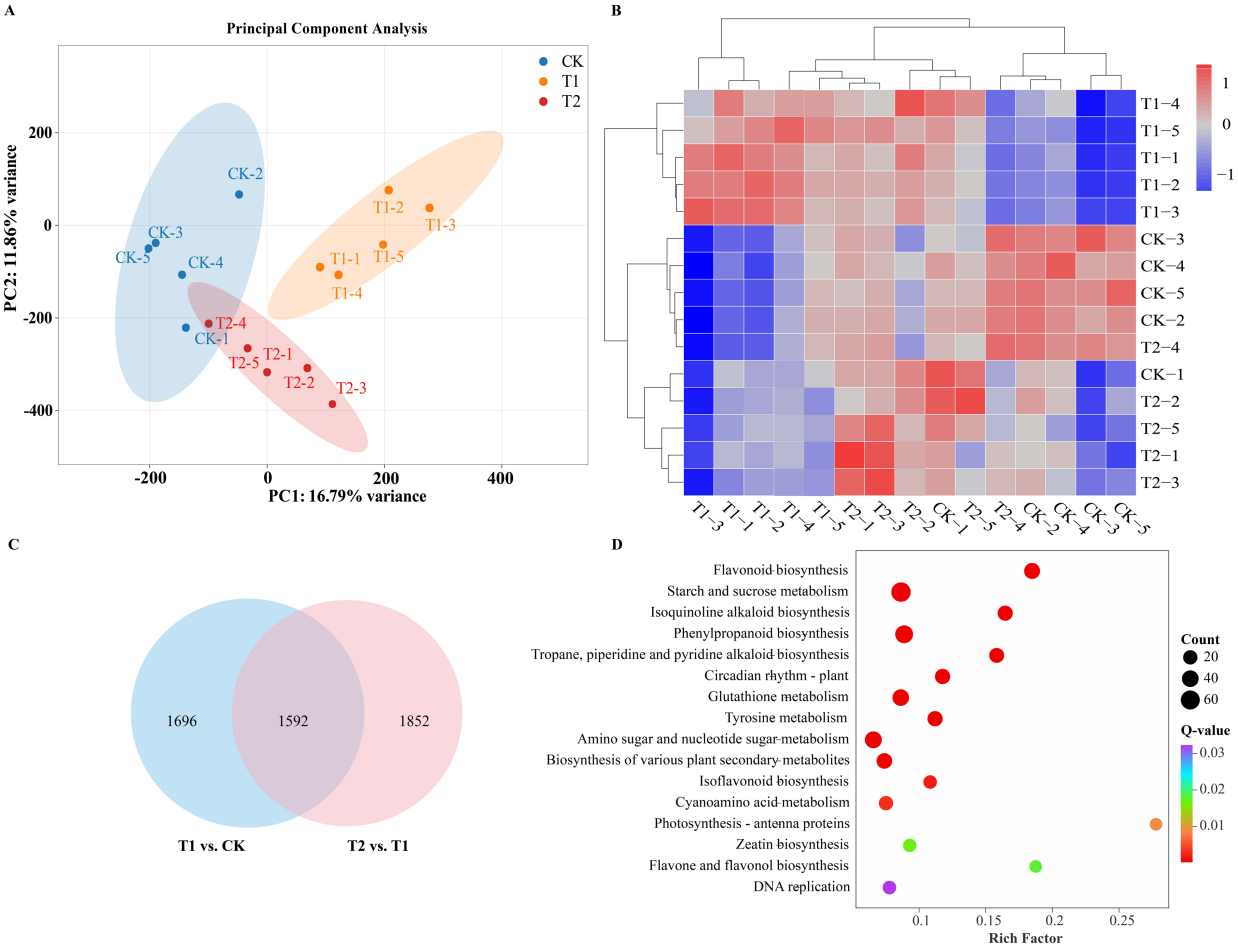
**(A)** Principal component analysis among three treatments in lily bulbs. **(B)** The correlation clustering heatmap of relative gene expression of three treatments in lily bulbs. **(C)** Venn diagram of genes expressed in the two comparison groups. **(D)** KEGG enrichment of the common DEGs in the two comparison groups.

A total of 144.97 Gb of clean reads were obtained after filtering, with 146.89 Gb of raw reads from the control and two treated samples. The Q20 and Q30 proportions of each sample surpassed 99.05 and 96.95%, respectively (Supplementary Table S4). A transcriptome database containing 91,837 unigenes (average length: 1,089 bp; N50: 1,419 bp; N90: 532 bp) was assembled using Trinity software. BUSCO assessment (*n* = 255) revealed 98.82% complete BUSCOs (C: 252 [S: 56, D: 196]), with minimal fragmentation (0.78%) and missing genes (0.39%), indicating high assembly completeness and could be used for subsequent bioinformatic analysis. All unigenes were searched against the KEGG, NR, Swiss-Prot, GO and Pfam databases, with 43,707, 56,001, 41,995, 49,332, and 46,314 annotated unigenes (Supplementary Table S5), respectively. Principal component analysis (PCA) and correlation clustering heatmaps of the 15 analyzed samples indicated that distinct clusters formed for different groups and that there were significant differences in gene expression between the T1 group and the other groups (Figures 4A,B). The DEGs for the different comparison groups were identified by DESeq on the basis of their FPKM values. A total of 3,288 DEGs were identified between T1 and CK (with 1,999 upregulated and 1,289 downregulated genes) (Supplementary Figure 7A, whereas the T2 vs. T1 comparison group contained 3,444 DEGs (with 1,902 upregulated and 1,542 downregulated genes) (Supplementary Figure 7B), and 1,592 DEGs were shared between the two comparison groups (Figure 4C). These results indicated that there was a significant change in gene expression in the lily bulbs among the different treatments. KEGG enrichment analysis of the shared DEGs revealed that they were enriched in 117 pathways. Among them, a total of 16 KEGG pathways showed significant enrichment, with the most abundant being “flavonoid biosynthesis,” followed by “starch and sucrose metabolism,” “phenylpropanoid biosynthesis,” and “isoquinoline alkaloid biosynthesis” (Figure 4D), demonstrating that the DEGs involved in the biosynthesis of secondary metabolites (flavonoids, phenolic acids, alkaloids, lignans, etc.) participate in the resistance responses of lily to *F. oxysporum*. Other significantly enriched pathways included “tropane, piperidine and pyridine alkaloid biosynthesis,” “circadian rhythm–plant,” “glutathione metabolism,” “tyrosine metabolism,” “amino sugar and nucleotide sugar metabolism,” and “biosynthesis of various plant secondary metabolites.” Moreover, the DEGs between the T1 and CK groups were specifically enriched in amino acid metabolism and lipid metabolism, whereas the DEGs between the T2 and T1 groups were uniquely enriched in plant hormone signal transduction (Supplementary Figures 7C,D).

**Figure 5:**
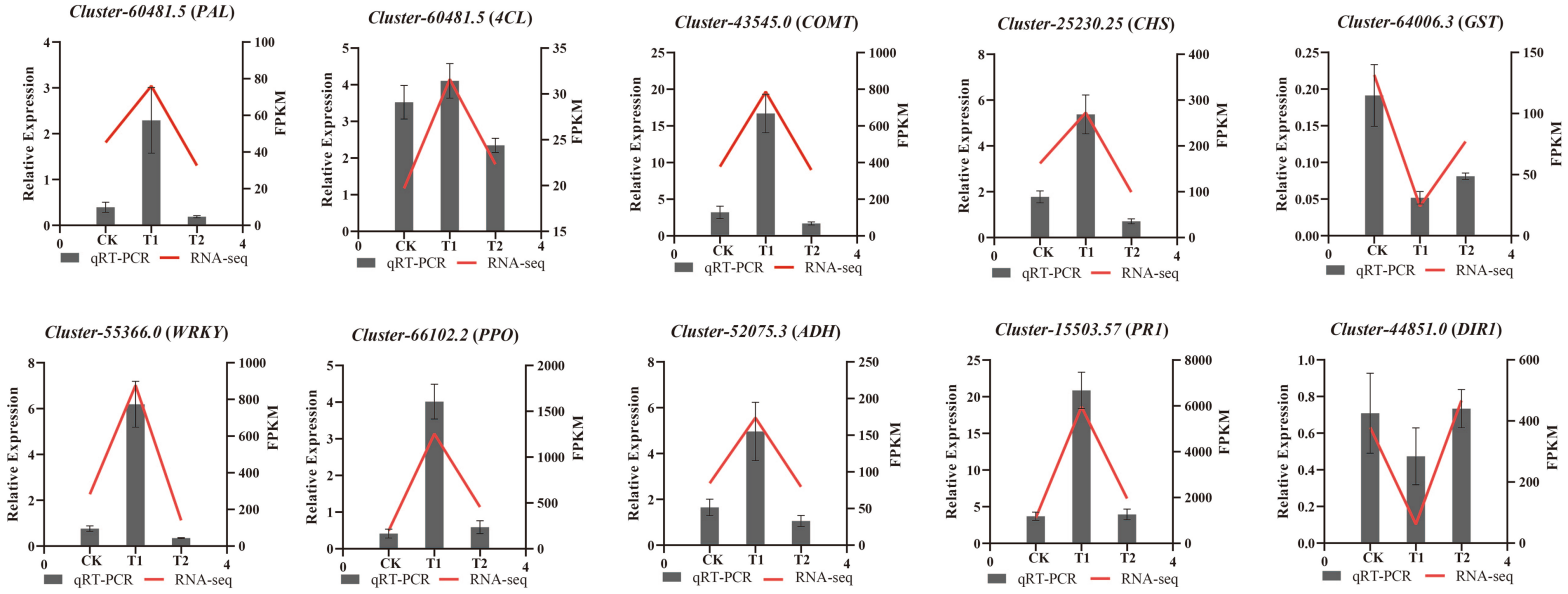
The relative expression levels of ten selected DEGs were compared by RNA-seq and RT-qPCR.

To validate the RNA-seq data, RT-qPCR analysis was conducted for 10 candidate genes involved in phenylpropanoid biosynthesis, flavonoid biosynthesis, isoquinoline alkaloid biosynthesis, glutathione metabolism, plant hormone signal transduction, and pathogenesis-related (PR) protein expression. As shown in Figure 5, the expression patterns of the selected genes were consistent with the RNA-seq data, highlighting the reliability of the transcriptome results.

### 3.7 Metabolomic analysis of lily bulbs treated with strain YFLYS026

**Figure 6:**
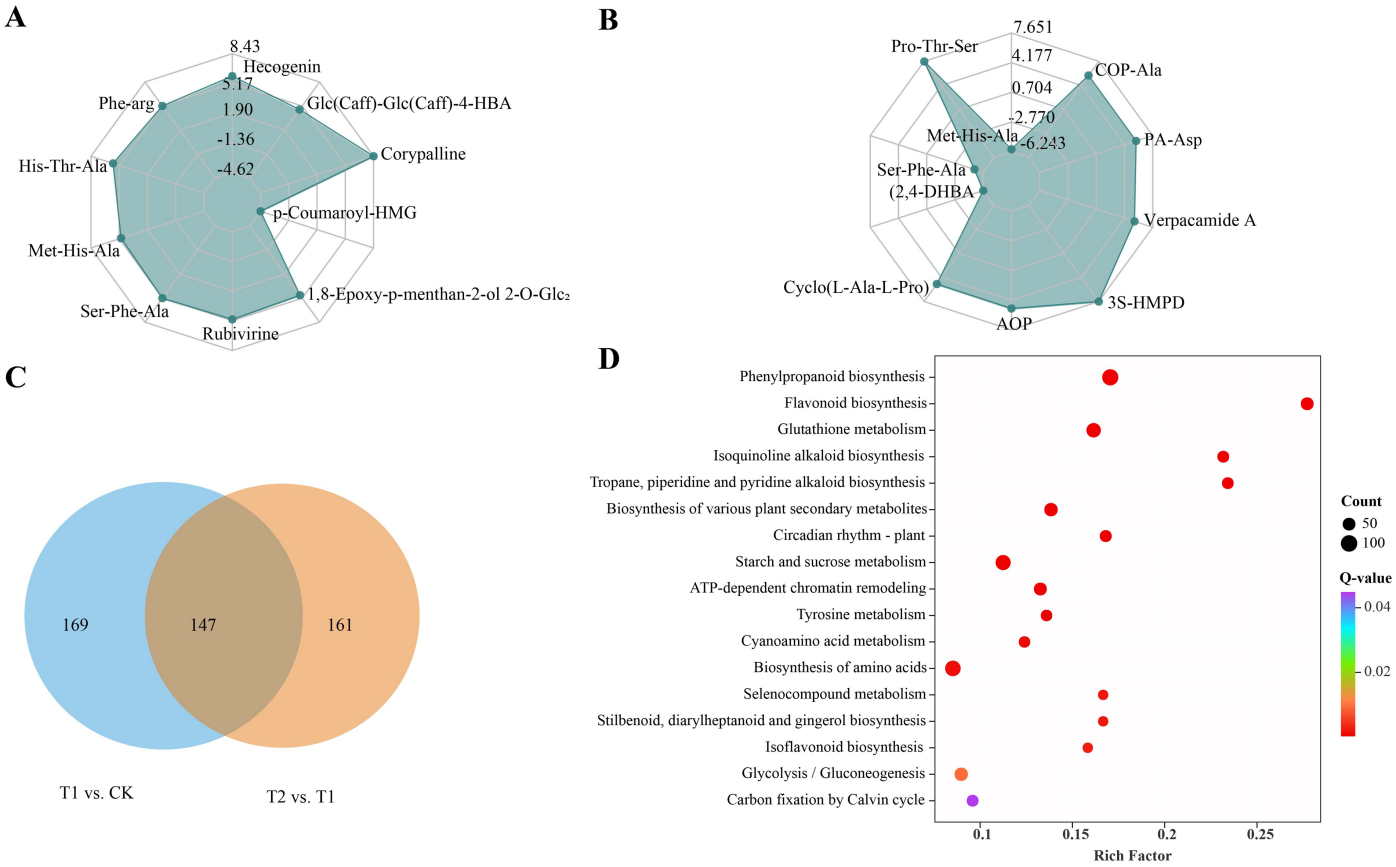
**(A)** The top 10 metabolites with the largest absolute value of log_2_FC were screened in T1 vs. CK. **(B)** The top 10 metabolites with the largest absolute value of log_2_FC were screened in T2 vs. T1. **(C)** Venn diagram showing DAMs numbers shared in each comparison group. **(D)** Top 20 enriched KEGG pathways of DAMs shared in each comparison group.

To gain an overall view of the metabolites in infected lily bulbs affected by these biocontrol agents, a UPLC–MS/MS-based widely targeted metabolomics analysis was performed to evaluate the metabolite profiles in detail. A total of 2,239 metabolites were obtained from all the samples and clearly separated into three groups according to the PCA results (Supplementary Figure 8). According to the results of the quantitative analyses of all the detected metabolites and the fold change threshold, a total of 316 DAMs were obtained between the T1 and CK groups, of which the levels of 232 metabolites were upregulated and 84 metabolites were downregulated (Supplementary Figure 9A); these DAMs included mainly phenolic acids, steroids, alkaloids, amino acids and derivatives (AADs), lipids and flavonoids (Supplementary Figure 9B). The top 10 DAMs with the greatest absolute log_2_FC values between the T1 and CK group, including one downregulated phenolic acid (p-Coumaroyl-HMG), one upregulated phenolic acid (Glc(Caff)-Glc(Caff)-HBA), four upregulated AADs (Phe-Arg, His-Thr-Ala, Ser-Phe-Ala and Met-His-Ala), two upregulated alkaloids (corypalline and rubivirine), one upregulated steroid (hecogenin), and one upregulated terpenoid (1,8-epoxy-p-menthan-2-ol 2-O-Glc₂) (Figure 6A). In the T2 vs. T1 group, there were 308 DAMs, including 107 whose levels were upregulated and 201 whose levels were downregulated (Supplementary Figure 9C), which were classified mainly as phenolic acids, AADs, alkaloids and terpenoids (Supplementary Figure 9D). The top 10 DAMs between T2 and T1 included two downregulated amino acids and derivatives (Met-His-Ala and Ser-Phe-Ala), one downregulated phenolic acid (2,4-DHBA), six upregulated AADs (Pro-Thr-Ser, COP-Ala, cyclo (L-Ala-L-Pro), AOP, 3S-HMPD, and PA-Asp), and one upregulated alkaloid (verpacamide A) (Figure 6B). A total of 147 DAMs overlapped between the different comparison groups, among which phenolic acid, alkaloid, and AAD DAMs were the most highly enriched, accounting for more than 12% (Figure 6C; Supplementary Figure 10A). Among them, 4 phenolic acids (4-HBA-Glc(2R,3S,4S,5R,6R)-ester, 2-(Glc-methoxy)-BA, p-Co-HMG, and 4-GlcO-BA), 3 alkaloids (5-MeO-DMT, veratraline B and 5-HCI-AP), and 3 AADs (Pro-Thr-Ser, O-Ac-Ser and Fru-Arg) were upregulated in the CK and T2 groups, whereas the other DAMs were upregulated in the T1 group (Supplementary Figures 10B–D). On the basis of the annotations of the shared DAMs, the associated KEGG pathways were determined; the analysis revealed that14 metabolites were significantly enriched in the top 10 metabolic pathways, including phenylpropanoid biosynthesis, the biosynthesis of various alkaloids, sulfur metabolism, propanoate metabolism, the cAMP signaling pathway, flavonoid biosynthesis, the sphingolipid signaling pathway, the cGMP-PKG signaling pathway, and the biosynthesis of various plant secondary metabolites (Figure 6D). Among these pathways, metabolites involved in the “phenylpropanoid biosynthesis” and “biosynthesis of various alkaloids” pathways showed the greatest accumulation, and may be involved in disease resistance in plant-pathogen interactions.

### 3.8 Combined metabolomics and transcriptomics analysis

**Figure 7:**
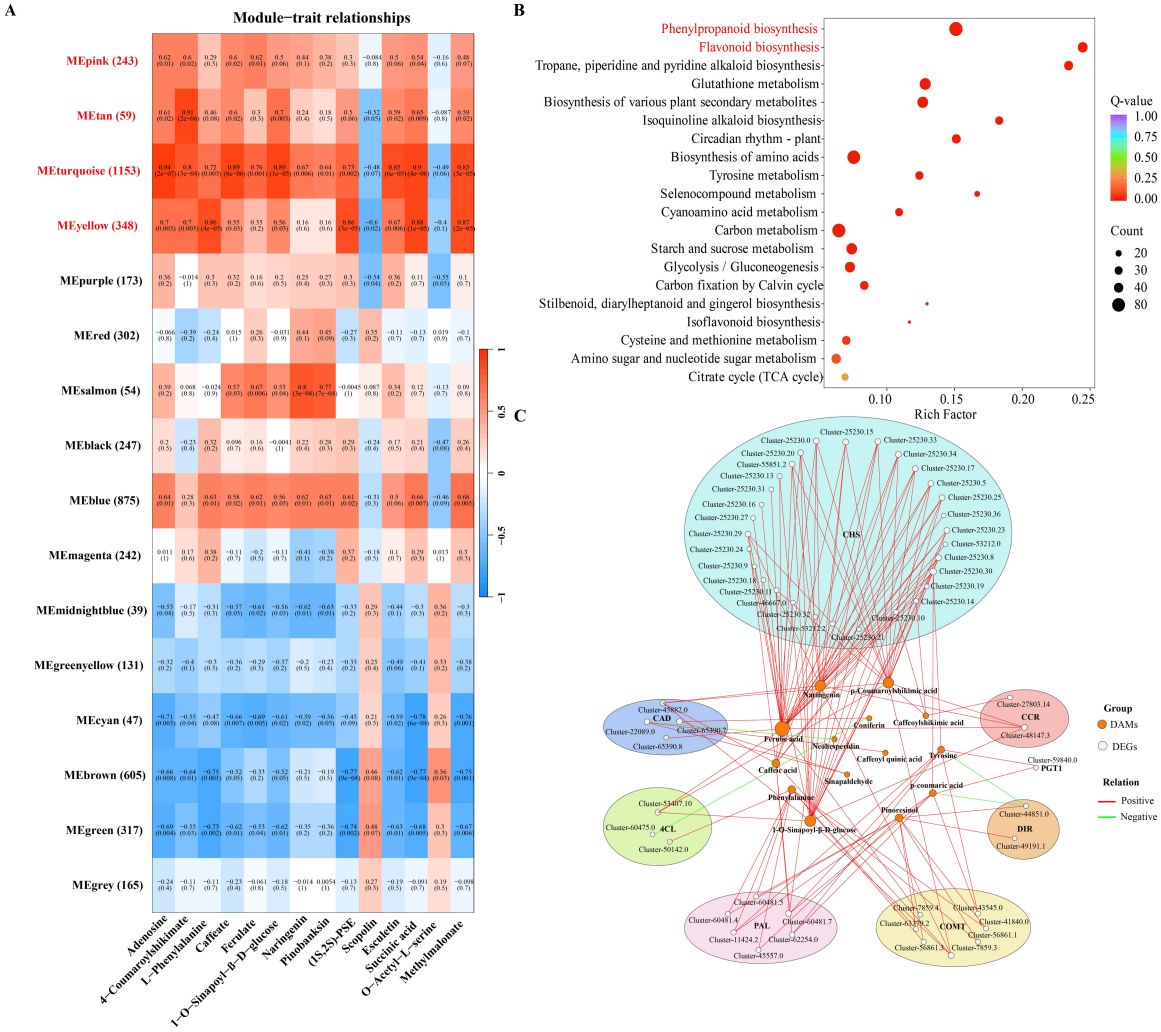
**(A)** Module-metabolite relationships. Each cell contains the corresponding correlation and *p*-value. **(B)** KEGG functional enrichment of DEGs in the MEpink, MEtan, MEturquoise, and MEyellow modules. **(C)** Phenylpropanoid and flavonoid transcriptional regulatory network based on Pearson correlation analysis.

**Figure 8:**
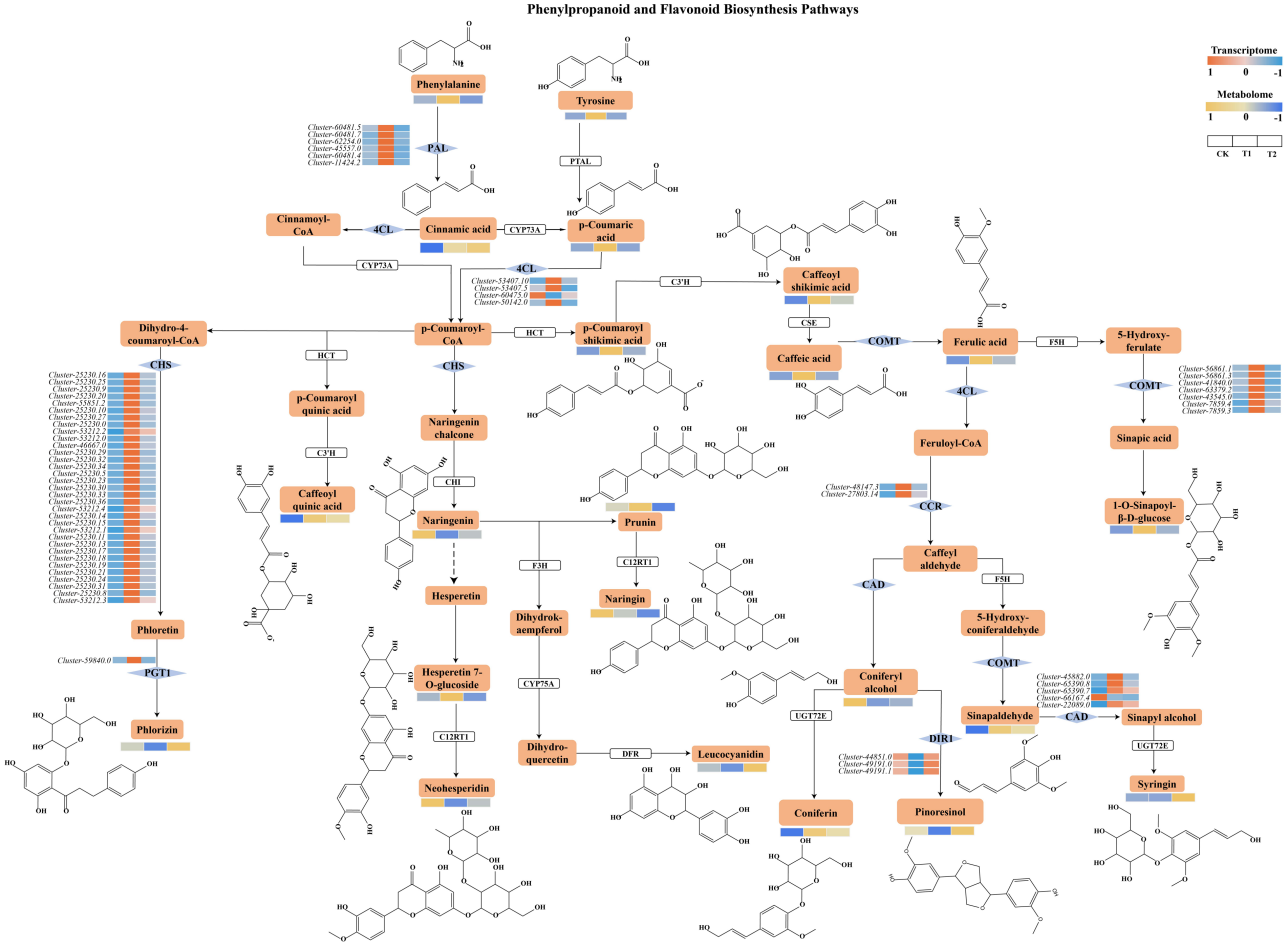
Expression analysis of genes and metabolites involved in phenylpropanoid and flavonoid biosynthesis pathways in all lily bulb samples.

KEGG pathway enrichment analysis identified 14 DAMs commonly shared between both comparison groups (adenosine, 4-coumaroylshikimate, L-phenylalanine, caffeate, ferulate, 1-O-sinapoyl-*β*-D-glucose, naringenin, pinobanksin, (1S, 2S)-PSE, scopolin, esculetin, succinic acid, O-acetyl-L-serine, and methylmalonate) that significantly enriched the top 10 metabolic pathways and were designated as core metabolites. A total of 45,401 genes were selected to construct the network after the removal of genes with very low expression (average TPM of <1). A soft threshold of β = 12 was selected to construct the coexpression modules, and hierarchical clustering revealed a total of 16 gene modules with strong coexpression relationships among the genes within each module (Supplementary Figures 11A,B). The MEpink, MEtan, MEturquoise, and MEyellow modules were significantly positively correlated with the contents of the core metabolites. To examine the biological functions of 1,803 genes in these four modules (Figure 7A), KEGG enrichment analysis was performed. According to the results, these modules were significantly enriched in phenylpropanoid and flavonoid metabolic processes (Figure 7B). Within the phenylpropanoid and flavonoid metabolic pathways, five of the 28 detected metabolites (cinnamic acid, phlorizin, syringin, pinoresinol, and leucocyanidin) exhibited increased accumulation following *B. pyrrocinia* inoculation. In contrast, except for coniferyl alcohol, naringin and neohesperidin, most metabolites exhibited greater accumulation in the T1 group. The correlation network analysis revealed coordinated regulation between phenylpropanoid and flavonoid metabolic genes and their corresponding metabolites (Figure 7C). The chalcone synthase (*CHS*) gene cluster exhibited strong positive correlations with flavonoid precursors including naringenin and p-coumaroylshikimic acid, whereas lignin biosynthesis-related gene clusters, such as cinnamyl alcohol dehydrogenase (*CAD*), phenylalanine ammonia-lyase (*PAL*), caffeic acid 3-O-methyltransferase (*COMT*), cinnamoyl-CoA reductase (*CCR*), 4-coumarate CoA ligase (*4CL*), dirigent protein (*DIR*), were associated with specific lignin monomers and intermediates such as phenylalanine, coniferin, sinapaldehyde, and pinoresinol. Notably, *DIR* genes showed specific positive correlations with pinoresinol, supporting their role in lignan biosynthesis, while ferulic acid served as a central hub connecting multiple gene clusters. Among these genes, multiple genes were downregulated in lily bulbs treated with strain YFLYS026, while only the *DIR* gene regulating the coupling of coniferyl alcohol to form lignans (pinoresinol) was upregulated (Figure 8). These findings suggested that strain YFLYS026 induced *DIR*-mediated pinoresinol accumulation and cell wall lignification to create a physical barrier against pathogens.

## 4 Discussion

Lily bulb rot, caused by *F. oxysporum*, is a typical destructive soil-borne disease that negatively affects lily production. Biological control has become an increasingly important means to inhibit plant diseases because it is a green and efficient approach. In particular, the application of specific BCAs is a viable alternative to chemical control for disease management. *Burkholderia* spp., an important bacterial genus, have been reported to have good inhibitory activity against plant pathogens and have proven to be important biocontrol strain candidates (Sandani et al., 2019). However, the application of the *Burkholderia* genus for the control of lily bulb rot has not yet been reported. In this study, YFLYS026 was isolated from the rhizosphere soil of healthy lily bulbs and was identified as *B. pyrrocinia* on the basis of morphological and phylogenetic analyses. Under controlled laboratory conditions, strain YFLYS026 exhibited distinct antagonistic activity on PDA medium, significantly inhibiting mycelial growth and exhibiting varying degrees of inhibitory effects on the growth of different pathogens, with the highest inhibition rates observed against *C. fructicola* (59.02%) and *F. oxysporum* (54.83%). Previous reports revealed that *B. pyrrocinia* can resist poplar canker caused by three pathogens (*Cytospora chrysosperma*, *Phomopsis macrospora*, and *Fusicoccum aesculi*) (Liu et al., 2020), control postharvest banana anthracnose caused by *C. fructicola* to preserve fruit quality (Zhu et al., 2024), inhibit the mycelial growth of *Magnaporthe oryzae and Aspergillus* spp. (Gong et al., 2019), and mitigate wheat scab caused by *F. graminearum* (Wang et al., 2024). The results indicated that *B. pyrrocinia* had broad-spectrum antifungal activity, highlighting its efficacy for the management of different plant diseases. Meanwhile, strain YFLYS026 had plant growth-promoting traits, including nitrogen fixation, phosphorus solubilization, and siderophore production. As we know, phosphorus is one of the essential macronutrients, along with nitrogen, required by plants for their survival and vital functions (Azaroual et al., 2020; Gong et al., 2019). Siderophores are a class of secondary metabolites synthesized by microbes and certain monocotyledonous plants in iron-deficient environments (Deb and Tatung, 2024). Microorganisms that produce siderophores can regulate the iron concentration in their surroundings by forming Fe^3+^-siderophore complexes, thereby preventing pathogenic microbes from obtaining the iron they need and inhibiting their growth and pathogenicity (Morales-Cedeño et al., 2021). Pyochelin, a siderophore produced by various *Pseudomonas* and *Burkholderia* species, has been reported to effectively inhibit banana anthracnose and *Fusarium* wilt (Zhu et al., 2024; Ho et al., 2021). In addition to siderophore, the volatile organic compounds (VOCs) produced by *B.pyrrocinia*, such as dimethyl disulfide (DMDS), may have potential as antifungal compounds (Farag et al., 2013; Gong et al., 2019; Liu et al., 2020). Strain YFLYS026 may also produce these antimicrobial compounds to enhance plant defense response against pathogens, but this topic requires further verification. The *in vivo* biocontrol efficacy of YFLYS026 against bulb rot was further assessed. Inoculation of lily bulbs with strain YFLYS026 significantly diminished disease symptoms in a pot experiment, with 61.11% biocontrol efficacy under greenhouse conditions. These findings reinforce that strain YFLYS026 could serve as a potential biocontrol resource.

Plant defense responses to biotic or abiotic stress are associated with large-scale changes in gene expression and metabolism. In this study, thousands of DEGs were found in the two comparison groups (T1 vs. CK and T2 vs. T1), and in both cases, the number of upregulated genes was higher than that of the downregulated genes, indicating that the lily bulb, via a complex network, activates plant immune responses and regulates the expression of defense-related genes. The common DEGs in the two comparison groups exhibited significant enrichment in multiple KEGG metabolic pathways, including flavonoid biosynthesis, starch and sucrose metabolism, phenylpropanoid biosynthesis, and isoquinoline alkaloid biosynthesis. Phenylpropanoid and flavonoid biosynthesis have been widely reported to protect against environmental stresses (Yadav et al., 2020; Li et al., 2021). Starch and sucrose are the main forms of energy storage and utilization in plants; when plants are infected by pathogens or subjected to environmental stress, the defense response consumes a large amount of energy, manifesting as bursts of reactive oxygen species and immune signaling triggered by pathogen-associated molecular patterns (Thibaud et al., 2004; Tarkowski et al., 2019). Starch and sucrose metabolism can quickly provide energy through pathways such as glycolysis and the tricarboxylic acid cycle, supporting the initiation and maintenance of plant defense mechanisms. For example, when wheat is infected by necrotrophic pathogens, the expression levels of starch metabolism-related genes change, which may be associated with energy reallocation to combat the pathogen (Ferreira et al., 2025). Furthermore, we also found that DEGs in the T2 vs. T1 comparison group were uniquely enriched in plant hormone signal transduction pathways. Plant immune circuits rely mainly on salicylic acid, jasmonic acid, and ethylene, which orchestrate distinct disease-resistance signaling networks (Bari and Jones, 2009). Hormone-triggered signal transduction alters ion flux across the membrane, eliciting a burst of cytosolic calcium; this calcium surge activates defense genes that reprogram secondary metabolism, channeling resources into phytoalexin production to block pathogen ingress (Boller and He, 2009). In plants, some compounds called phytoalexins are synthesized and accumulate to maintain normal growth and development in response to biotic or abiotic stress (Zaynab et al., 2018). Most phytoalexins, such as flavonoids, terpenoids, alkaloids, and phenolic acids, can be synthesized via the phenylpropanoid metabolism pathway (Ahuja et al., 2012). Among the top 10 DAMs, there was a relatively high number of increased AADs in the two comparison groups, especially in the T2 vs. T1 group, and accounting for more than 12% of the shared AADs, of which Pro-Thr-Ser, O-acetyl-L-serine and arginine were increased in the T2 treatment group compared with the T1 group. Amino acids are required for the synthesis of proteins, including disease-responsive proteins (Hildebrandt et al., 2015). Arginine, as a mobile nitrogen depot in many plants, can be converted into polyamines (PAs) or nitric oxide once mobilized. PAs can affect protein synthesis and improve plant disease resistance (Majerus et al., 2007), while NO taps intracellular Ca^2+^ stores, switching on antioxidant and defense genes that limit oxidative injury (Shi et al., 2010). AADs also can facilitate carbon shuttling. Coenzyme A, synthesized from *β*-alanine through the pantothenate pathway, is an essential cofactor in metabolic processes, modulating plant growth and increasing stress tolerance (Ouyang et al., 2025). The increased accumulation of AADs in the lily bulb treated with *B. pyrrocinia* likely provided abundant nitrogen nutrition to the roots, facilitating AAD synthesis and degradation, and creating favorable conditions for plant growth following microbial colonization.

Integrated transcriptomic and metabolomic analysis is commonly employed to identify functional genes and elucidate underlying metabolic pathways (Mao et al., 2021; Zhang et al., 2024). In this study, we performed WGCNA and found that the DEGs in the coexpression modules closely related to the 14 DAMs (MEpink, MEtan, MEturquoise, and MEyellow) were significantly enriched in the phenylpropanoid and flavonoid metabolic pathways. The phenylpropanoid metabolism-based defense response to pathogens has been partially characterized in plants (Meng et al., 2021; Yadav et al., 2020). Flavonoid biosynthesis is an important pathway downstream of phenylpropanoid metabolism, whose protective functions against environmental stresses have been widely reported (Yadav et al., 2020). As the main products of phenylpropanoid metabolism, phenolics and flavonoids can disrupt the plasma membrane of pathogens, impair mitochondria function, and inhibit cell wall formation, all of which have detrimental effects on pathogen growth (Aboody and Mickymaray, 2020; Dong and Lin, 2021). In this study, we found that when the lily bulbs were infected with the pathogen, *F. oxysporum* induced the upregulation of most genes, such as *PAL*, *4CL*, *COMT*, *CCR*, *PGT1* and *CHS,* but after the effect of *B. pyrrocinia*, only the expression level of *DIR* was increased. The activities of most genes encoding phenylpropanoid and flavonoid biosynthesis enzymes, such as *PAL*, *4CL*, *COMT*, *CCR*, *PGT1* and *CHS*, increased significantly in the bulbs after infection with *F. oxysporum,* which was similar to the findings of previous studies on lilies (Deng et al., 2024; He et al., 2019). *DIR* is a lipid transfer protein that plays a crucial role in plant defense signaling by facilitating the movement of lipid-based molecules between different parts of the plant, which is essential for systemic acquired resistance (Champigny et al., 2013; Chen et al., 2024). *DIR* aids in the long-distance signaling of defense-related molecules such as azelaic acid (AzA), dehydroabietinal (DA), glycerol-3-phosphate (G3P), and methyl salicylate (MeSA). These molecules are vital for amplifying defense signals in distant tissues, ensuring that the entire plant can respond to pathogenic attack (Carella et al., 2017). The coordinated action of these signals enables the plant to effectively combat and resist subsequent pathogen attacks, thereby improving its overall resilience and health. Studies indicate that the *DIR* gene is conserved across various plant species, including citrus, and is expressed in response to pathogen infection and other stress conditions, underscoring its role in the defense mechanisms of citrus plants (Duan et al., 2024). Pearson correlation analysis revealed that the downregulation of the key enzymes involved in phenylpropanoid and flavonoid metabolic pathways was positively correlated with the decreased accumulation of some DAMs, and only five compounds (cinnamic acid, phlorizin, syringin, leucocyanidin and pinoresinol) were increased. Cinnamic acid is a toxic phenolic acid with antifungal activity against Sclerotinia stem rot caused by *S. sclerotiorum* (Romagnoli et al., 2022; Wang et al., 2019). The growth and spore germination of multiple fungi were significantly inhibited by treatment with syringic acid at different concentrations (Shaik et al., 2016). Lignans are vital physiological, developmental, and ecological plant metabolites. Plants’ dirigent protein crucially regulates the initial coupling reactions that form lignans, pinoresinol-lariciresinol reductase *(PLR)* then encode successive reduction reactions to form lariciresinol and secoisolariciresinol from pinoresinol. Pinoresinol is a phenylpropanoid dimer formed by *DIR*-mediated oxidative coupling of two coniferyl alcohol molecules, which has been reported to defend against pathogens as phytoalexins. For instance, *GmDIR22* was identified as a regulator of coniferyl alcohol coupling to form the lignan (+)-pinoresinol to restrict the hyphal growth of *Phytophthora sojae* in soybean (Markulin et al., 2019). In pea pods, the aromatic compound pinoresinol monoglucoside were detected on the endocarp epidermal cell layer of pea pod infected by *Fusarium solani f.* sp. *Phaseoli* (Seneviratne et al., 2015). Thus, the alleviation of lily bulb rot disease may be attributed to YFLYS026 inducing *DIR* to promote lignin accumulation, thereby strengthening the physical barrier to prevent pathogen infection.

## 5 Conclusion

In summary, strain YFLYS026, isolated from the rhizosphere soil surrounding healthy lily bulbs, was identified as *B. pyrrocinia* based on morphological identification, physiobiochemical characteristics and phylogenetic analysis. Strain YFLYS026 exhibited a broad antifungal spectrum and secreted siderophores that chelate free iron ions in the environment, creating iron-limited conditions to inhibit pathogen growth. In pot experiment, strain YFLYS026 could effectively prevented the infection of lily bulbs by *F. oxysporum*, with a biocontrol efficacy of 61.11%. Moreover, after inoculation of strain YFLYS026, *DIR* expression was induced and upregulated in lily bulbs, driving pinoresinol (lignin monomer) accumulation, promoting host cell wall lignification, and forming a physical barrier to block pathogen invasion. The integrated multi-omics analysis showed that phenylpropanoid and flavonoid metabolic pathways were downregulated, potentially indicating carbon metabolic flux redistribution to prioritize lignin synthesis. These results underscore that *B. pyrrocinia* YFLYS026 is a promising biocontrol candidate for managing *Fusarium*-induced soil-borne diseases.

## Data Availability

The datasets presented in this study can be found in online repositories. The names of the repository/repositories and accession number(s) can be found in the article/Supplementary material.

## References

[ref1] Aboody M. S. A. Mickymaray S. (2020). Anti-fungal efficacy and mechanisms of flavonoids. Antibiotics (Basel, Switzerland) 9:45. doi: 10.3390/antibiotics9020045, 31991883 PMC7168129

[ref3] Ahuja I. Kissen R. Bones A. M. (2012). Phytoalexins in defense against pathogens. Trends Plant Sci. 17, 73–90. doi: 10.1016/j.tplants.2011.11.002, 22209038

[ref4] Aulia Rahma A. Suryanti Somowiyarjo S. Joko T. (2020). Induced disease resistance and promotion of shallot growth by *Bacillus velezensis* B-27. Pak. J. Biol. Sci. 23, 1113–1121. doi: 10.3923/pjbs.2020.1113.1121 32981242

[ref5] Azaroual S. E. Hazzoumi Z. Mernissi N. E. Aasfar A. Meftah Kadmiri I. Bouizgarne B. (2020). Role of inorganic phosphate solubilizing Bacilli isolated from Moroccan phosphate rock mine and rhizosphere soils in wheat (*Triticum aestivum* L) phosphorus uptake. Curr. Microbiol. 77, 2391–2404. doi: 10.1007/s00284-020-02046-8, 32468184

[ref6] Bach E. Passaglia L. M. P. Jiao J. Gross H. (2022). *Burkholderia* in the genomic era: from taxonomy to the discovery of new antimicrobial secondary metabolites. Crit. Rev. Microbiol. 48, 121–160. doi: 10.1080/1040841X.2021.1946009, 34346791

[ref7] Bari R. Jones J. D. (2009). Role of plant hormones in plant defence responses. Plant Mol. Biol. 69, 473–488. doi: 10.1007/s11103-008-9435-0, 19083153

[ref8] Boller T. He S. Y. (2009). Innate immunity in plants: an arms race between pattern recognition receptors in plants and effectors in microbial pathogens. Science 324, 742–744. doi: 10.1126/science.1171647, 19423812 PMC2729760

[ref9] Carella P. Kempthorne C. J. Wilson D. C. Isaacs M. Cameron R. K. (2017). Exploring the role of DIR1, DIR1-like and other lipid transfer proteins during systemic immunity in *Arabidopsis*. Physiol. Mol. Plant Pathol. 97, 49–57. doi: 10.1016/j.pmpp.2016.12.005

[ref10] Champigny M. J. Isaacs M. Carella P. Faubert J. Fobert P. R. Cameron R. K. (2013). Long distance movement of DIR1 and investigation of the role of DIR1-like during systemic acquired resistance in Arabidopsis. Front. Plant Sci. 4:230. doi: 10.3389/fpls.2013.00230, 23847635 PMC3701462

[ref11] Chen R. Yu J. Yu L. Xiao L. Xiao Y. Chen J. . (2024). The ERF transcription factor LTF1 activates DIR1 to control stereoselective synthesis of antiviral lignans and stress defense in Isatis indigotica roots. Acta Pharm. Sin. B 14, 405–420. doi: 10.1016/j.apsb.2023.08.011, 38261810 PMC10792966

[ref12] Das S. Dutta S. Ghosh S. Mukherjee A. (2024). Chitinolytic microorganisms for biological control of plant pathogens: a comprehensive review and meta-analysis. Crop Prot. 185:106888. doi: 10.1016/j.cropro.2024.106888

[ref13] Deb C. R. Tatung M. (2024). Siderophore producing bacteria as biocontrol agent against phytopathogens for a better environment: a review. S. Afr. J. Bot. 165, 153–162. doi: 10.1016/j.sajb.2023.12.031

[ref14] Deng J. Che X. Gu Y. Qu Y. Liu D. (2024). Integrated multi-omics investigation revealed the importance of phenylpropanoid metabolism in the defense response of *Lilium regale* Wilson to fusarium wilt. Hortic. Res. 11:uhae140. doi: 10.1093/hr/uhae140, 38988612 PMC11233880

[ref15] Dong N. Q. Lin H. X. (2021). Contribution of phenylpropanoid metabolism to plant development and plant-environment interactions. J. Integr. Plant Biol. 63, 180–209. doi: 10.1111/jipb.13054, 33325112

[ref16] Duan M. Bao L. Eman M. Han D. Zhang Y. Zheng B. . (2024). The ectopic expression of the *MpDIR1(t)* gene enhances the response of plants from *Arabidopsis thaliana* to biotic stress by regulating the defense genes and antioxidant flavonoids. Plants 13:2692. doi: 10.3390/plants13192692, 39409562 PMC11478391

[ref17] Esmaeel Q. Pupin M. Kieu N. P. Chataigné G. Béchet M. Deravel J. . (2016). *Burkholderia* genome mining for nonribosomal peptide synthetases reveals a great potential for novel siderophores and lipopeptides synthesis. Microbiolopen 5, 512–526. doi: 10.1002/mbo3.347, 27060604 PMC4906002

[ref18] Farag M. A. Zhang H. Ryu C.-M. (2013). Dynamic chemical communication between plants and bacteria through airborne signals: induced resistance by bacterial volatiles. J. Chem. Ecol. 39, 1007–1018. doi: 10.1007/s10886-013-0317-9, 23881442 PMC3738840

[ref19] Ferreira L. C. Santana F. M. Scagliusi S. M. M. Beckmann M. Mur L. A. J. (2025). Omic characterisation of multi-component defences against the necrotrophic pathogen Pyrenophora tritici-repentis in wheat. Plant Biol. (Stuttg.) 27, 347–361. doi: 10.1111/plb.13746, 39918991 PMC11950905

[ref20] Fontana D. C. de Paula S. Torres A. G. de Souza V. H. M. Pascholati S. F. Schmidt D. . (2021). Endophytic Fungi: biological control and induced resistance to Phytopathogens and abiotic stresses. Pathogens (Basel, Switzerland) 10:570. doi: 10.3390/pathogens10050570, 34066672 PMC8151296

[ref21] Gong A. Zhu Z. Lu Y. Wan H. Wu N. Dimuna C. . (2019). Functional analysis of *Burkholderia pyrrocinia* WY6-5 on phosphate solubilizing, antifungal and growth-promoting activity of maize. Sci. Agric. Sin. 52, 1574–1586. doi: 10.3864/j.issn.0578-1752.2019.09.009

[ref22] He X. Li W. Zhang W. Jin X. Shenkute A. G. Aynalem T. . (2019). Transcriptome sequencing analysis provides insights into the response to *Fusarium oxysporum* in *Lilium pumilum*. Evol. Bioinformatics Online 15:1176934319838818. doi: 10.1177/1176934319838818, 31223231 PMC6563521

[ref23] Heo A. Y. Koo Y. M. Choi H. W. (2022). Biological control activity of plant growth promoting Rhizobacteria *Burkholderia contaminans* AY001 against tomato *Fusarium* wilt and bacterial speck diseases. Biology 11:619. doi: 10.3390/biology11040619, 35453817 PMC9028202

[ref24] Hildebrandt T. M. Nunes Nesi A. Araújo W. L. Braun H. P. (2015). Amino acid catabolism in plants. Mol. Plant 8, 1563–1579. doi: 10.1016/j.molp.2015.09.005, 26384576

[ref25] Ho Y. N. Hoo S. Y. Wang B. W. Hsieh C. T. Lin C. C. Sun C. H. . (2021). Specific inactivation of an antifungal bacterial siderophore by a fungal plant pathogen. ISME J. 15, 1858–1861. doi: 10.1038/s41396-020-00871-0, 33619352 PMC8163733

[ref26] Huang Y. Wang H. Li J. Shan X. Liu J. Yan L. (2025). Use of *Pseudomonas lurida* QNF3 as a biocontrol agent to control postharvest apple ring rot. Postharvest Biol. Technol. 232:113953. doi: 10.1016/j.postharvbio.2025.113953

[ref27] Jiang Y. Liang Q. Wei L. Meng X. Lin K. Yue Y. (2021). Effect of several biological pesticides and their mixtures on the prevention and control of bulb rot disease during the storage period of Lanzhou lily. Chin. J. Biol. Control 37, 1041–1049. doi: 10.16409/j.cnki.2095-039x.2021.06.002

[ref9001] Katoh K. Standley D. M. (2013). MAFFT multiple sequence alignment software version 7: improvements in performance and usability. Molecular biology and evolution 30, 772–780. doi: 10.1093/molbev/mst010, 23329690 PMC3603318

[ref28] Jiao X. Zhang X. Zhou Y. (2021). Advances in the study of medicinal Lilium bulb diseases. China Plant Prot. 41, 30–37.

[ref29] Langfelder P. Horvath S. (2008). WGCNA: an R package for weighted correlation network analysis. BMC Bioinformatics 9:559. doi: 10.1186/1471-2105-9-559, 19114008 PMC2631488

[ref30] Li P. Ruan Z. Fei Z. Yan J. Tang G. (2021). Integrated transcriptome and metabolome analysis revealed that flavonoid biosynthesis may dominate the resistance of *Zanthoxylum bungeanum* against stem canker. J. Agric. Food Chem. 69, 6360–6378. doi: 10.1021/acs.jafc.1c00357, 34043342

[ref31] Liu M. Philp J. Wang Y. Hu J. Wei Y. Li J. . (2022). Plant growth-promoting rhizobacteria *Burkholderia vietnamiensis* B418 inhibits root-knot nematode on watermelon by modifying the rhizosphere microbial community. Sci. Rep. 12:8381. doi: 10.1038/s41598-022-12472-2, 35589885 PMC9120051

[ref32] Liu A. Zhang P. Bai B. Bai F. Jin T. Ren J. (2020). Volatile organic compounds of Endophytic *Burkholderia pyrrocinia* strain JK-SH007 promote disease resistance in poplar. Plant Dis. 104, 1610–1620. doi: 10.1094/PDIS-11-19-2366-RE, 32271644

[ref2] Luo S. Tian C. Zhang H. Yao Z. Guan Z. Li Y. . (2023). Isolation and Identification of Biocontrol Bacteria against Atractylodes Chinensis Root Rot and Their Effects. Microorganisms 11:2384. doi: 10.3390/microorganisms11102384, 37894042 PMC10609459

[ref33] Majerus V. Bertin P. Lutts S. (2007). Effects of iron toxicity on osmotic potential, osmolytes and polyamines concentrations in the African rice (*Oryza glaberrima* Steud.). Plant Sci. 173, 96–105. doi: 10.1016/j.plantsci.2007.04.003

[ref34] Mao J. Huang L. Chen M. Zeng W. Feng Z. Huang S. . (2021). Integrated analysis of the transcriptome and metabolome reveals genes involved in Terpenoid and flavonoid biosynthesis in the loblolly pine (*Pinus taeda* L.). Front. Plant Sci. 12:729161. doi: 10.3389/fpls.2021.729161, 34659295 PMC8519504

[ref35] Markulin L. Corbin C. Renouard S. Drouet S. Gutierrez L. Mateljak I. . (2019). Pinoresinol-lariciresinol reductases, key to the lignan synthesis in plants. Planta 249, 1695–1714. doi: 10.1007/s00425-019-03137-y, 30895445

[ref36] Meng L. Zhang X. Wang L. Liu H. Zhao Y. Yi K. . (2021). Transcriptome profiling unveils the mechanism of phenylpropane biosynthesis in rhizome development of Caucasian clover. PLoS One 16:e0254669. doi: 10.1371/journal.pone.0254669, 34255805 PMC8277049

[ref37] Morales-Cedeño L. R. Orozco-Mosqueda M. D. C. Loeza-Lara P. D. Parra-Cota F. I. de Los Santos-Villalobos S. Santoyo G. (2021). Plant growth-promoting bacterial endophytes as biocontrol agents of pre- and post-harvest diseases: fundamentals, methods of application and future perspectives. Microbiol. Res. 242:126612. doi: 10.1016/j.micres.2020.126612, 33059112

[ref38] Naqvi S. A. H. Farhan M. Ahmad M. Kiran R. Shahbaz M. Abbas A. . (2025). Fungicide resistance in Fusarium species: exploring environmental impacts and sustainable management strategies. Arch. Microbiol. 207:31. doi: 10.1007/s00203-024-04219-6, 39792175

[ref39] O’Donnell K. Ward T. J. Robert V. A. R. G. Crous P. W. Geiser D. M. Kang S. (2015). DNA sequence-based identification of *Fusarium*: current status and future directions. Phytoparasitica 43, 583–595. doi: 10.1007/s12600-015-0484-z

[ref40] Ouyang N. Hu W. Meng J. Wang B. (2025). Amino acid derivatives as regulatory molecules: mechanisms in plant growth and stress tolerance. Crop J. 13, 668–680. doi: 10.1016/j.cj.2025.04.005

[ref41] Romagnoli R. Oliva P. Prencipe F. Manfredini S. Germanò M. P. De Luca L. . (2022). Cinnamic acid derivatives linked to arylpiperazines as novel potent inhibitors of tyrosinase activity and melanin synthesis. Eur. J. Med. Chem. 231:114147. doi: 10.1016/j.ejmech.2022.114147, 35114540

[ref42] Sandani H. B. P. Ranathunge N. P. Lakshman P. L. N. Weerakoon W. M. W. (2019). Biocontrol potential of five *Burkholderia* and *Pseudomonas* strains against *Colletotrichum truncatum* infecting chilli pepper. Biocontrol Sci. Tech. 29, 727–745. doi: 10.1080/09583157.2019.1597331

[ref43] Schmittgen T. D. Livak K. J. (2008). Analyzing real-time PCR data by the comparative C(T) method. Nat. Protoc. 3, 1101–1108. doi: 10.1038/nprot.2008.73, 18546601

[ref44] Seneviratne H. K. Dalisay D. S. Kim K. W. Moinuddin S. G. Yang H. Hartshorn C. M. . (2015). Non-host disease resistance response in pea (*Pisum sativum*) pods: biochemical function of DRR206 and phytoalexin pathway localization. Phytochemistry 113, 140–148. doi: 10.1016/j.phytochem.2014.10.013, 25457488

[ref45] Shaik A. B. Ahil S. B. Govardhanam R. Senthi M. Khan R. Sojitra R. . (2016). Antifungal effect and protective role of Ursolic acid and three phenolic derivatives in the Management of Sorghum Grain Mold under Field Conditions. Chem. Biodivers. 13, 1158–1164. doi: 10.1002/cbdv.201500515, 27447843

[ref46] Shi J. Fu X. Z. Peng T. Huang X. S. Fan Q. J. Liu J. H. (2010). Spermine pretreatment confers dehydration tolerance of citrus in vitro plants via modulation of antioxidative capacity and stomatal response. Tree Physiol. 30, 914–922. doi: 10.1093/treephys/tpq030, 20462936

[ref47] Tang Y.-C. Liu Y.-J. He G.-R. Cao Y.-W. Bi M.-M. Song M. . (2021). Comprehensive analysis of secondary metabolites in the extracts from different lily bulbs and their antioxidant ability. Antioxidants 10:1634. doi: 10.3390/antiox10101634, 34679768 PMC8533310

[ref48] Tarkowski Ł. P. de Van Poel B. Höfte M. den Van Ende W. (2019). Sweet immunity: inulin boosts resistance of lettuce (*Lactuca sativa*) against grey mold (*Botrytis cinerea*) in an ethylene-dependent manner. Int. J. Mol. Sci. 20:1052. doi: 10.3390/ijms20051052, 30823420 PMC6429215

[ref49] Thibaud M. C. Gineste S. Nussaume L. Robaglia C. (2004). Sucrose increases pathogenesis-related PR-2 gene expression in *Arabidopsis thaliana* through an SA-dependent but NPR1-independent signaling pathway. Plant physiology and biochemistry: PPB 42, 81–88. doi: 10.1016/j.plaphy.2003.10.012, 15061088

[ref50] Tu J. Zhao X. Yang Y. Yi Y. Wang H. Wei B. . (2023). Two Bacillus spp. strains improve the structure and diversity of the rhizosphere soil microbial community of Lilium brownii var. viridulum. Microorganisms 11:1229. doi: 10.3390/microorganisms11051229, 37317201 PMC10223157

[ref51] Wang D. Luo W. Z. Zhang D. D. Li R. Kong Z. Q. Song J. . (2023). Insights into the biocontrol function of a *Burkholderia gladioli* strain against Botrytis cinerea. Microbiol. Spectrum 11:e0480522. doi: 10.1128/spectrum.04805-22, 36861984 PMC10101029

[ref52] Wang D. D. Nie J. J. Zhao R. B. Lu J. Wei Y. X. Yu L. . (2024). A novel *Burkholderia pyrrocinia* strain effectively inhibits *Fusarium graminearum* growth and deoxynivalenol (DON) production. Pest Manag. Sci. 80, 4883–4896. doi: 10.1002/ps.8200, 38817082

[ref53] Wang Y. Sun Y. Wang J. Zhou M. Wang M. Feng J. (2019). Antifungal activity and action mechanism of the natural product Cinnamic acid against *Sclerotinia sclerotiorum*. Plant Dis. 103, 944–950. doi: 10.1094/PDIS-08-18-1355-RE, 30895869

[ref54] Yadav V. Wang Z. Wei C. Amo A. Ahmed B. Yang X. . (2020). Phenylpropanoid pathway engineering: an emerging approach towards plant defense. Pathogens (Basel, Switzerland) 9:312. doi: 10.3390/pathogens9040312, 32340374 PMC7238016

[ref55] Yang L. Fu Y. Fan J. Huang Y. (2019). Morphological characteristics and karyotype analysis of three local varieties of *Lilium brownii* var.*viridulum*. Plant Sci. J. 37, 559–568. doi: 10.11913/PSJ.2095-0837.2019.50559

[ref56] Yin J. Bai R. Yuan L. Huang J. G. (2023). Application of Ceriporia lacerata HG2011 as biocontrol agent against multiple phytopathogenic fungi and oomycetes. Pestic. Biochem. Physiol. 190:105316. doi: 10.1016/j.pestbp.2022.105316, 36740332

[ref57] Zaman N. R. Chowdhury U. F. Reza R. N. Chowdhury F. T. Sarker M. Hossain M. M. . (2021). Plant growth promoting endophyte *Burkholderia contaminans* NZ antagonizes phytopathogen *Macrophomina phaseolina* through melanin synthesis and pyrrolnitrin inhibition. PLoS One 16:e0257863. doi: 10.1371/journal.pone.0257863, 34591915 PMC8483353

[ref58] Zaynab M. Fatima M. Abbas S. Sharif Y. Umair M. Zafar M. H. . (2018). Role of secondary metabolites in plant defense against pathogens. Microb. Pathog. 124, 198–202. doi: 10.1016/j.micpath.2018.08.034, 30145251

[ref59] Zhang L. Cao Y. Jin T. Lu S. Ren J. (2025). Effect of *ssuA* gene knockout on the colonization and biocontrol function of the biocontrol bacterium *Burkholderia pyrrocinia* JK-SH007. Tree Health 2, 55–71.

[ref60] Zhang Y. Cao X. Liu Q. Chen Y. Wang Y. Cong H. . (2024). Multi-omics analysis of *Streptomyces djakartensis* strain MEPS155 reveal a molecular response strategy combating *Ceratocystis fimbriata* causing sweet potato black rot. Food Microbiol. 122:104557. doi: 10.1016/j.fm.2024.104557, 38839221

[ref61] Zhao K. Penttinen P. Zhang X. Ao X. Liu M. Yu X. . (2014). Maize rhizosphere in Sichuan, China, hosts plant growth promoting *Burkholderia cepacia* with phosphate solubilizing and antifungal abilities. Microbiol. Res. 169, 76–82. doi: 10.1016/j.micres.2013.07.003, 23932330

[ref62] Zhu Y. Zhou E. Shu C. Cheng B. Liu X. Tang X. . (2024). Biocontrol of *Colletotrichum fructicola* in the postharvest Banana fruit using the Siderophore-producing strain BX1. J. Agric. Food Chem. 72, 22132–22143. doi: 10.1021/acs.jafc.4c04726, 39316703

