# Supplementary material for: Characteristics of Burkholderia pyrrocinia and the biocontrol mechanism by which it affects lily bulb rot caused by F. oxysporum
Source: Front Microbiol. 2026 Apr 23;17:1788193. doi: 10.3389/fmicb.2026.1788193 (PMC13149453; doi:10.3389/fmicb.2026.1788193)
Supplement: Supplementary file 2 [file Table_2.docx]

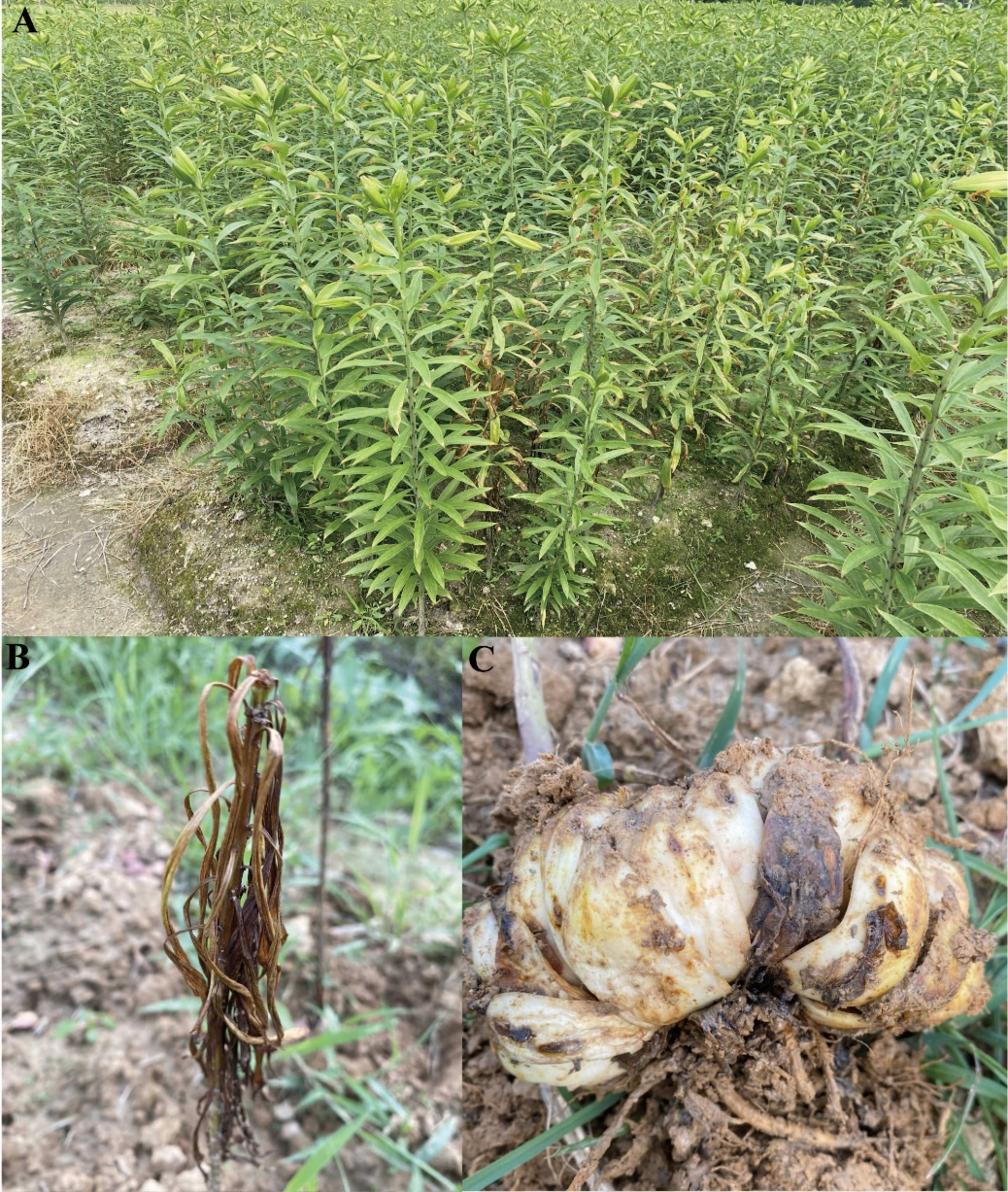


**Supplementary Figures.1** (A) The overall growth condition of lily plants in the fields of Wanzai County. (B) Aerial parts of diseased lily plants. (C) Bulb browning of diseased lily plants.


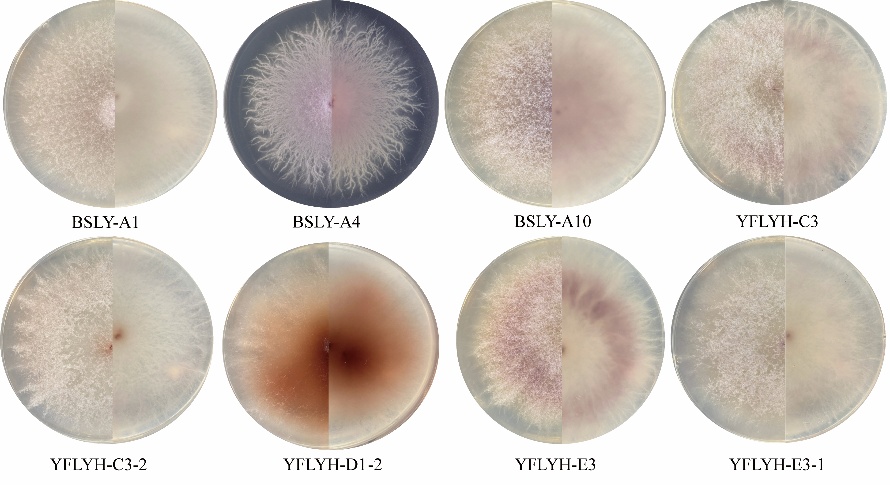


**Supplementary Figures 2** Eight pathogenic fungi isolated from the infected lily bulbs.


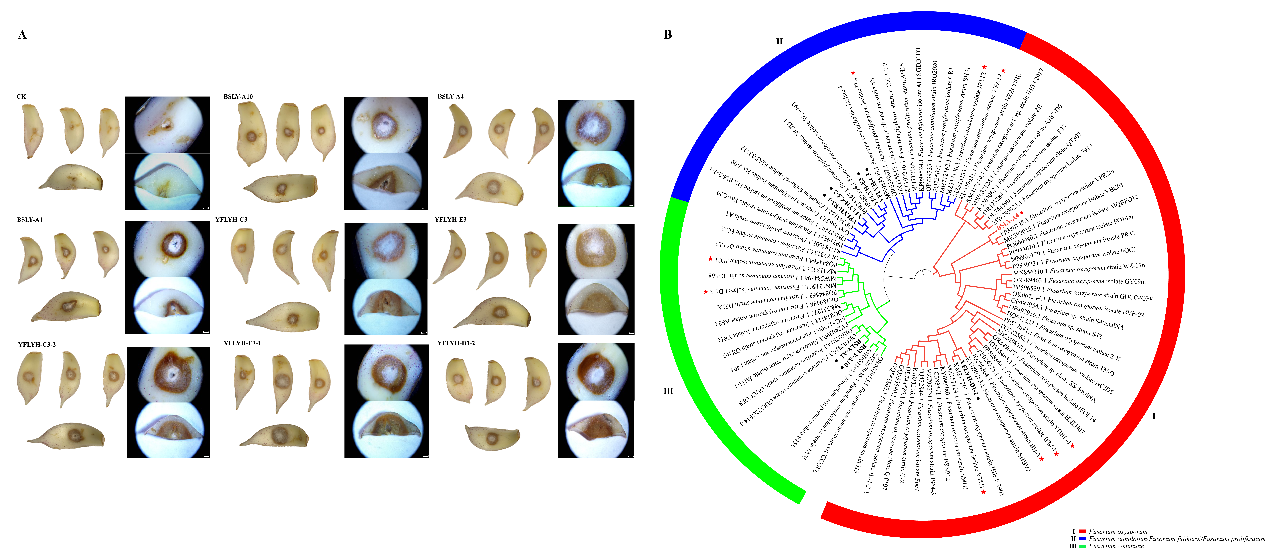


**Supplementary Figures 3 (A)** Disease symptoms of bulb rot in lilies treated with the different *Fusarium* species. **(B)** Phylogenetic tree of eight pathogens based on ITS sequence. “*” represented strains that have been reported. The red tree-like lines represented *F. oxysporum* group, the blue tree-like lines represented *F. proliferatum/F. annulatum/F. fujikuroi* group, the green tree-like lines represented *F. commune* group.


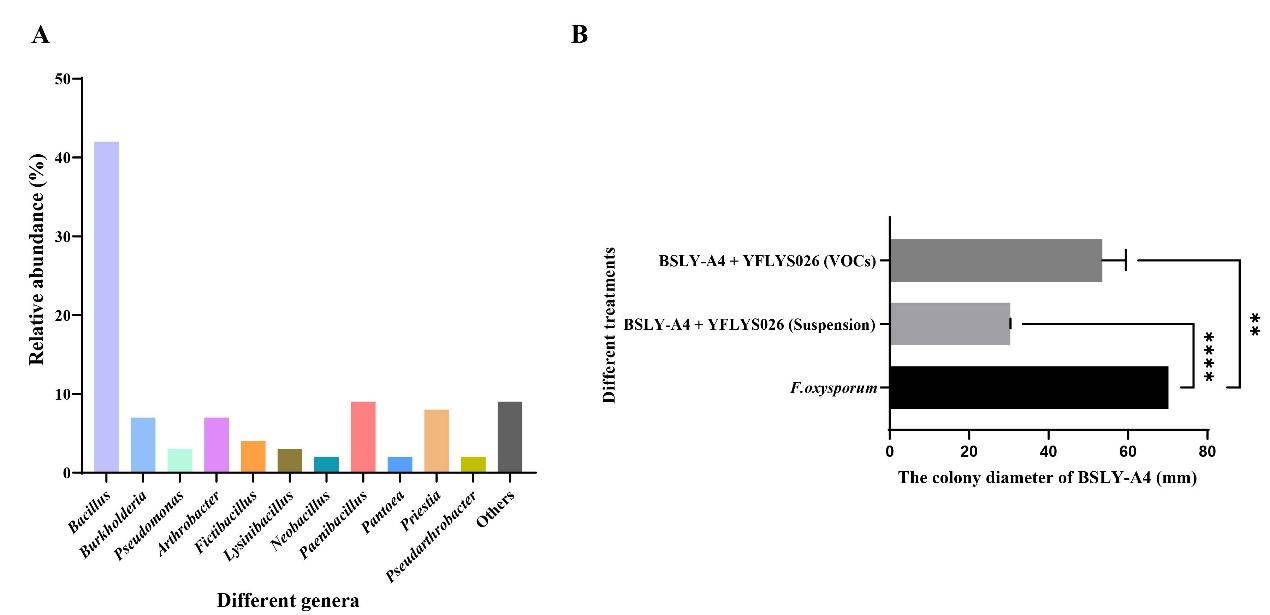


**Supplementary Figures 4 (A)** The relative abundance of several bacterial genera isolated from the rhizosphere soil of healthy lily bulbs. **(B)** The inhibitory effect of strain YFLYS026 on the mycelial growth of *F. oxysporum*.


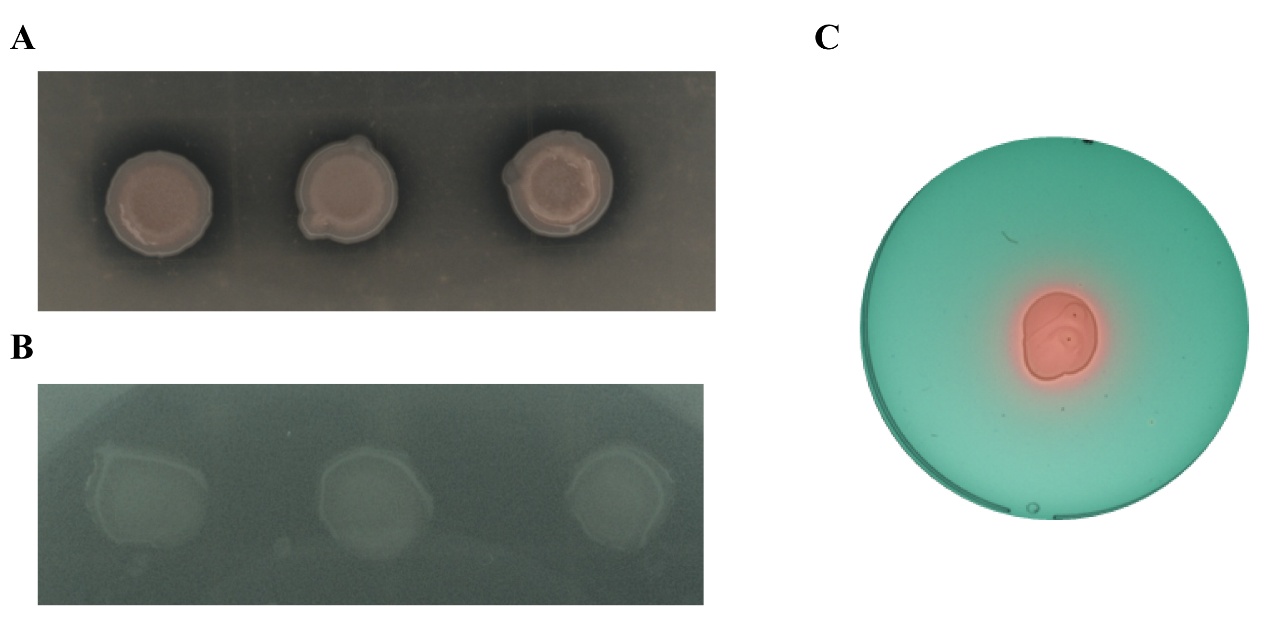


**Supplementary Figures 5 (A)** The growth characteristic of strain YFLYS026 on PVK medium. **(B)** The growth characteristic of strain YFLYS026 on ashby solid medium. **(C)** The growth characteristic of strain YFLYS026 on CAS medium.


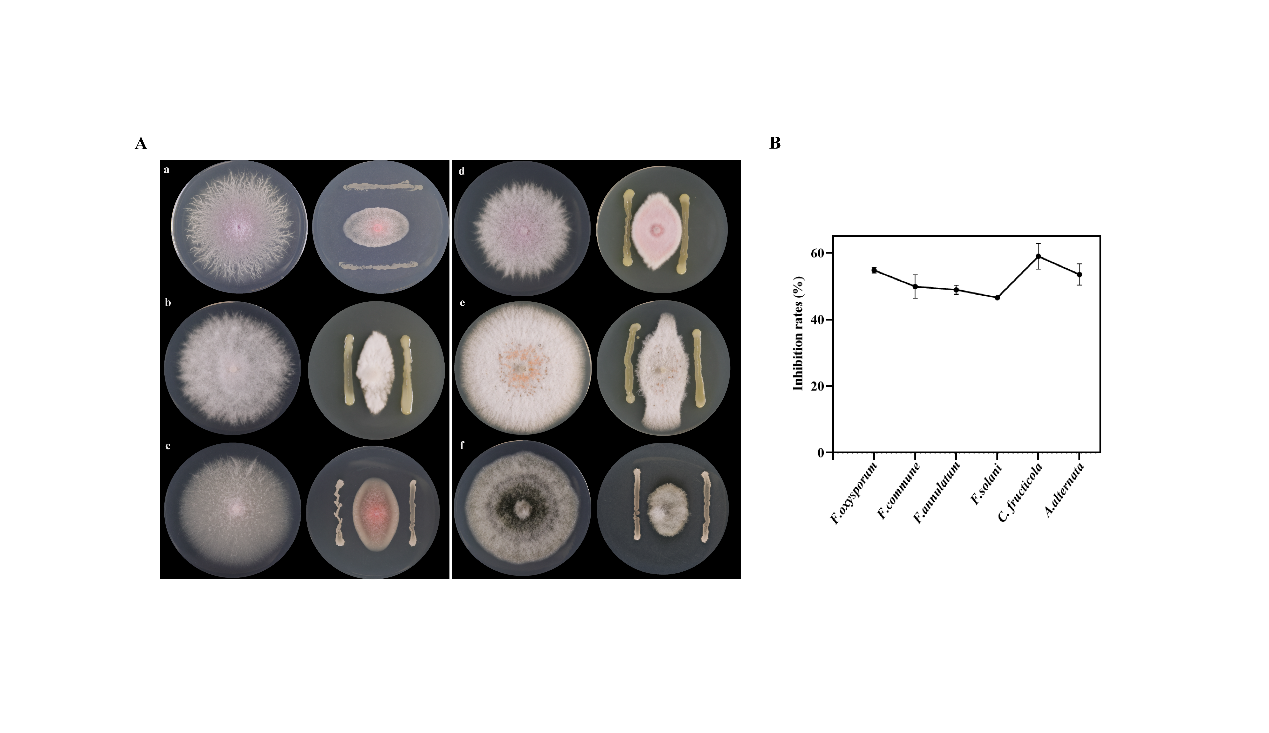


**Supplementary Figures 6 (A)** In vitro antifungal experiment of strain YFLYS026.a-f: *F. oxysporum*, *F. commune*, *F. annulatum, F. solani, A. alternata, C. fructicola.* **(B)** The inhibition rate of strain YFLYS026 against six pathogenic fungi.


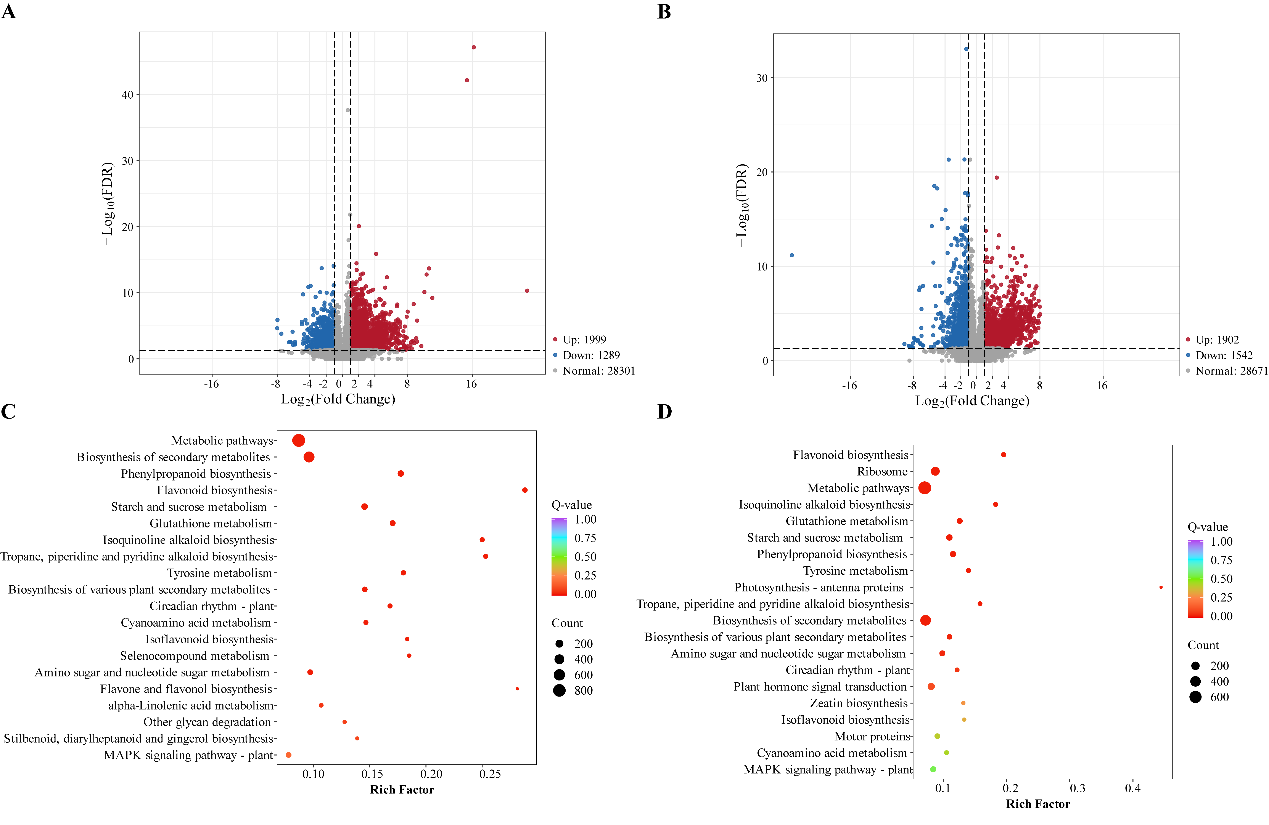


**Supplementary Figures 7** Number and KEGG enrichment of DEGs in the different comparison groups. (A) (C) T1 vs. CK. (B) (D) T2 vs. T1.


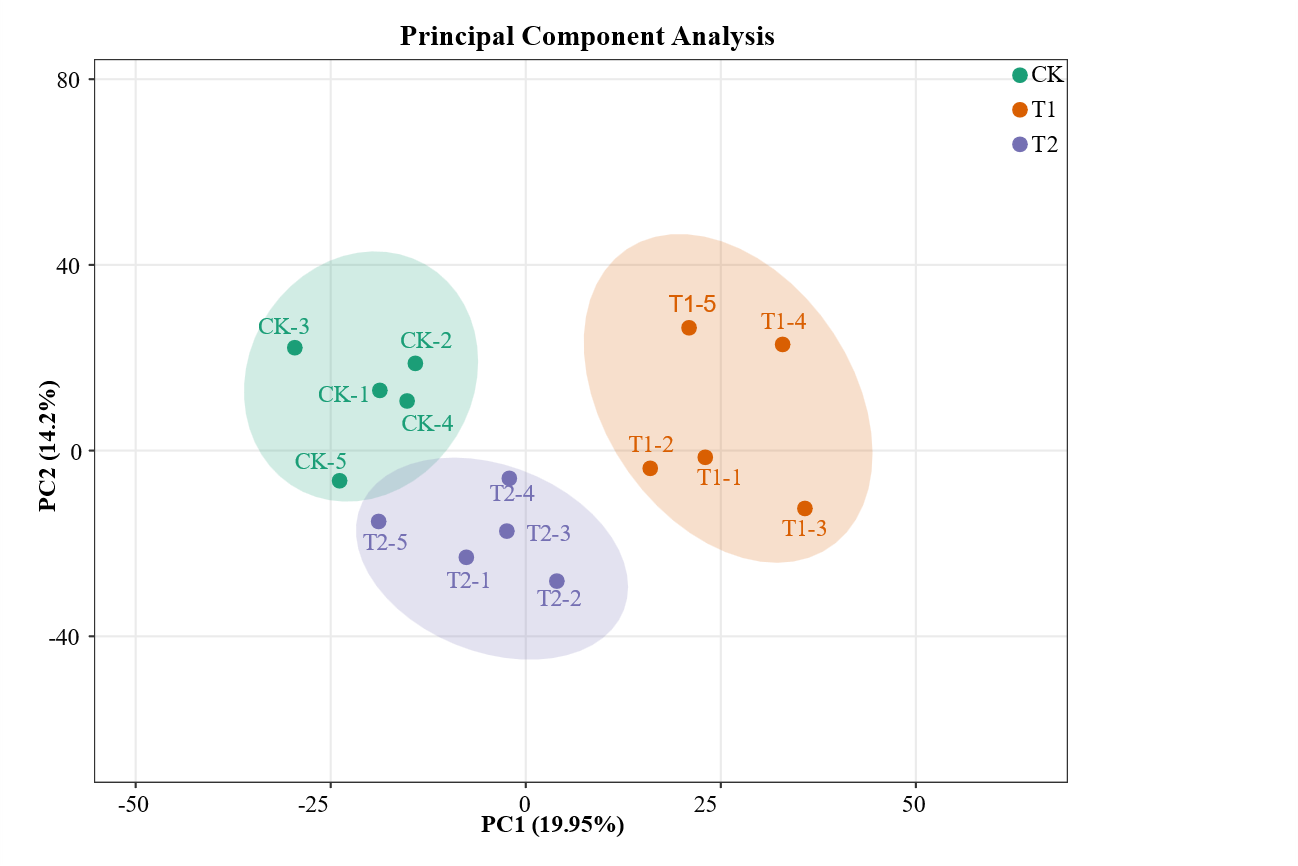


**Supplementary Figures 8** Principal component analysis of metabolites identified among three treatments in lily bulbs.


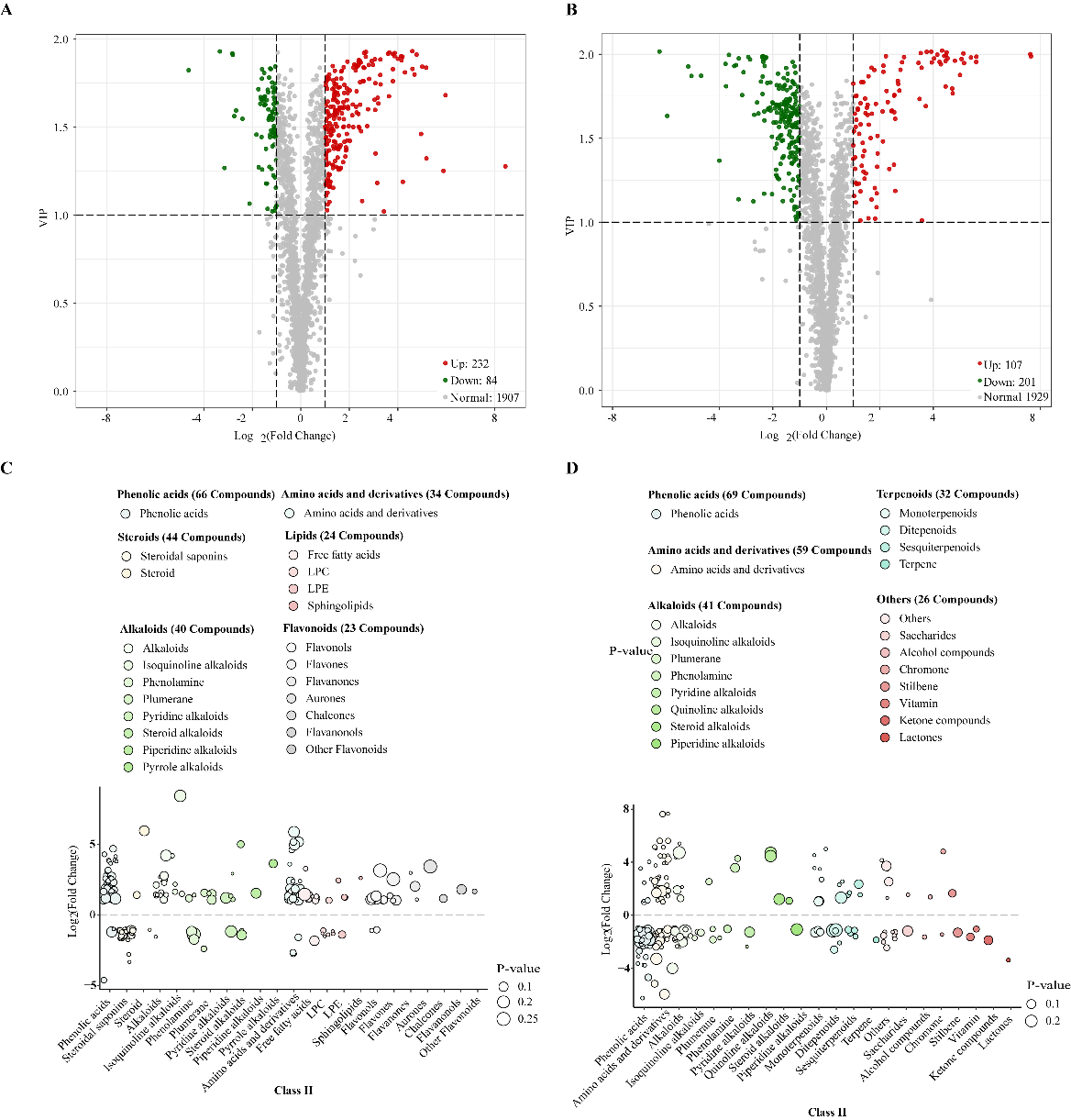


**Supplementary Figures 9** **(A)** Volcano plot of metabolites in T1 vs. CK. **(B)** Volcano plot of metabolites in T2 vs. T1. **(C)** Classification of all DAMs in T1 vs. CK. **(D)** Classification of all DAMs in T2 vs. T1.


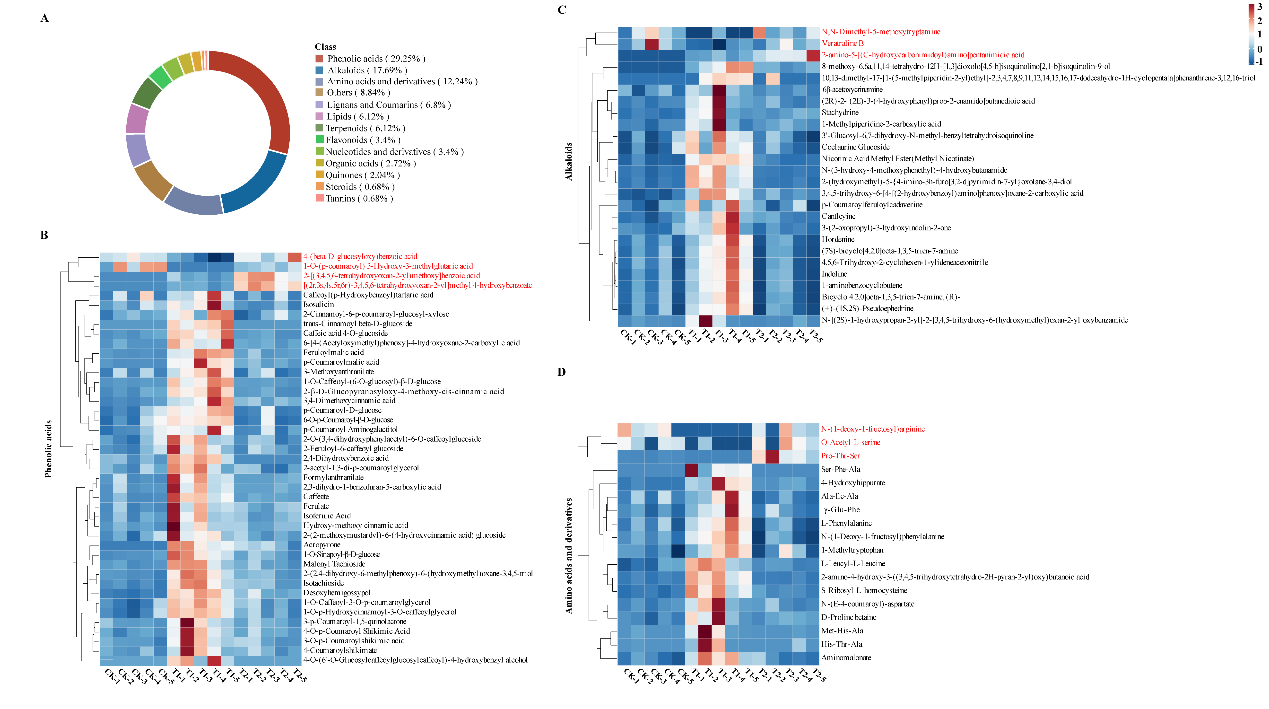


**Supplementary Figures 10** **(A)** Circular classification diagram of the common DAMs in the two comparison groups. **(B)** The heatmap of the common phenolic acids in the two comparison groups. **(C)** The heatmap of the common alkaloids in the two comparison groups. **(D)** The heatmap of the common amino acids and derivatives in the two comparison groups.


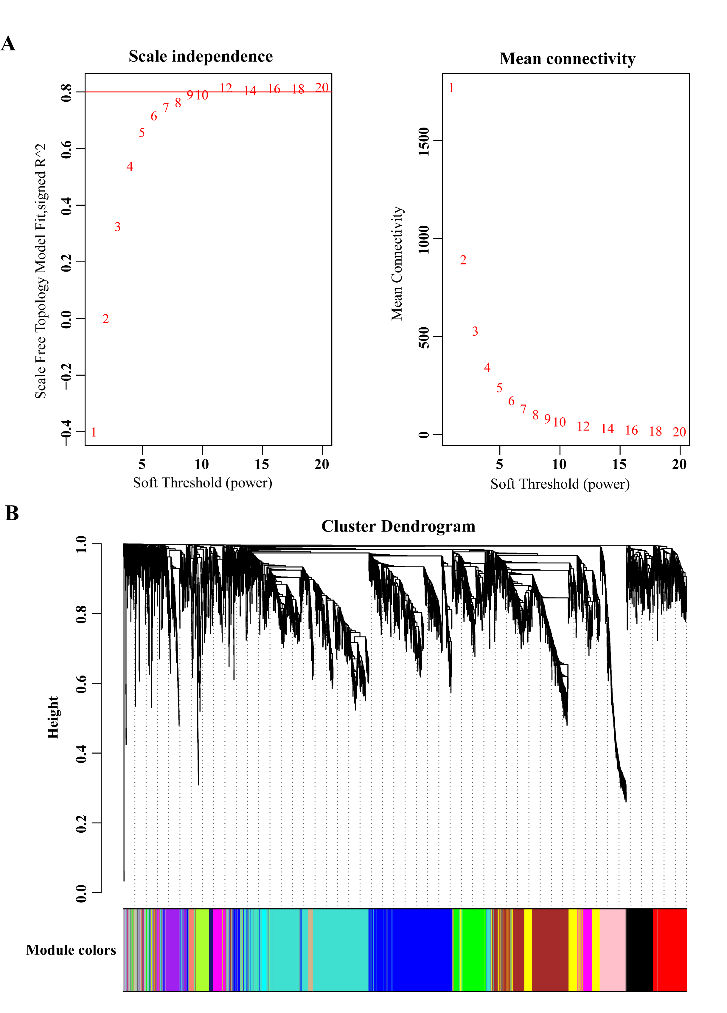


**Supplementary Figures 11** **(A)** Determination of the power beta value based on the adjacency matrix using WGCNA. **(B)** Dendrogram showing co-expression modules (clusters) identified by WGCNA.
